# Dissecting the Molecular Mechanism of Nucleotide-Dependent Activation of the KtrAB K^+^ Transporter

**DOI:** 10.1371/journal.pbio.1002356

**Published:** 2016-01-15

**Authors:** Andras Szollosi, Ricardo S. Vieira-Pires, Celso M. Teixeira-Duarte, Rita Rocha, João H. Morais-Cabral

**Affiliations:** 1 IBMC, Instituto de Biologia Molecular e Celular, Universidade do Porto, Porto, Portugal; 2 Instituto de Investigação e Inovação em Saúde, Universidade do Porto, Porto, Portugal; University of Zurich, SWITZERLAND

## Abstract

KtrAB belongs to the Trk/Ktr/HKT superfamily of monovalent cation (K^+^ and Na^+^) transport proteins that closely resemble K^+^ channels. These proteins underlie a plethora of cellular functions that are crucial for environmental adaptation in plants, fungi, archaea, and bacteria. The activation mechanism of the Trk/Ktr/HKT proteins remains unknown. It has been shown that ATP stimulates the activity of KtrAB while ADP does not. Here, we present X-ray structural information on the KtrAB complex with bound ADP. A comparison with the KtrAB-ATP structure reveals conformational changes in the ring and in the membrane protein. In combination with a biochemical and functional analysis, we uncover how ligand-dependent changes in the KtrA ring are propagated to the KtrB membrane protein and conclude that, despite their structural similarity, the activation mechanism of KtrAB is markedly different from the activation mechanism of K^+^ channels.

## Abstract 2

This study reveals how structural changes triggered by the exchange of bound ADP for ATP activate KtrAB, a potassium ion transporter involved in osmotic adaption in bacteria.

## Author Summary

Animals have organs that regulate the balance of water and ions in the fluids bathing their cells. In contrast, the cells of plants, bacteria, and fungi have little or no control over those fluids and, thus, they have to cope with changes in the local environment. These cells have therefore evolved specific molecular systems that are crucial for environmental adaptation. We study the molecular properties of the membrane protein KtrAB—a member of the Trk/Ktr/HKT superfamily of transport proteins that shuffle K^+^ and Na^+^ ions across the plasma membrane, closely resemble K^+^ channels, and underlie environmental adaptation of cells of plants, fungi, bacteria, and archaea. KtrAB is formed by the KtrB membrane protein and the KtrA cytosolic ring protein. KtrA binds to both ADP and ATP, resulting in a low-activity ADP-bound state and a high-activity ATP-bound state, respectively. We determined a low resolution structure of a low-activity form of the transport protein. A comparison of this structure with the structure of ATP-bound KtrAB reveals changes in both the KtrA ring and the KtrB membrane protein. We uncover how changes in the KtrA ring are propagated to KtrB and conclude that, despite their structural similarity, the activation mechanism of KtrAB is markedly different from the activation mechanism of K^+^ channels.

## Introduction

KtrAB belongs to the Trk/Ktr/HKT superfamily of monovalent cation (K^+^ and Na^+^) transport proteins that are found ubiquitously in nonanimal cells [1–3]. The Trk/Ktr/HKT superfamily comprises uniporters (K^+^ or Na^+^) and symporters (K^+^/Na^+^ or K^+^/H^+^) and underlies a plethora of cellular functions in plants, fungi, archaea, and bacteria such as K^+^ and Na^+^ uptake, regulation of cellular electrical activity, turgor compensation, osmotic adjustment (thereby contributing to resistance to drought and salinity), intra- and intercellular ion transport, motor cellular functions, and adjustment of membrane potential [2,4].

The structure of the KtrAB complex from the bacterium *Bacillus subtilis* was recently determined [5]. This protein complex plays an important role in the osmotic adaptation mechanism of this bacterium [6]. It is composed by a homodimeric KtrB membrane protein (GenBank: KIX81591.1) assembled with the KtrA octameric ring (GenBank: KIX81590.1). KtrB is responsible for ion permeation; each KtrB subunit has the architecture of a potassium channel pore domain [1,5,7] (S1A–S1D Fig), consisting of four M1-P-M2 (transmembrane helix 1-pore helix and loop- transmembrane helix 2) structural repeats (D1 to D4), which embrace an ion-permeable pore. KtrA binds ATP and adenosine diphosphate (ADP) and is responsible for regulation of KtrAB activity [5,8]. This cytosolic protein is a regulate conductance of K^+^ (RCK) domain [5,9–11] that assembles as an octameric ring, closely resembling the RCK gating rings of the MthK [12,13], BK [14–16], and GsuK [17] potassium channels (S1E–S1F Fig). The architecture of the KtrAB complex is also present in the TrkHA complex, another member of the Trk/Ktr/HKT superfamily [18].

Importantly, KtrB displays structural features that are not present in K^+^ channels. In particular, repeat D3 of KtrB has an insertion of ~10 residues that form an intramembrane loop pointing into the cytosolic pore. This intramembrane loop is also found in other members of the Trk/Ktr/HKT superfamily [1,7,19], and together with a highly conserved arginine residue (R417 in KtrB from *B*. *subtilis*), it obstructs the ion pathway of KtrAB and TrkHA [5,18,20]. The intramembrane loop and the conserved arginine are thought to be a central feature of the activation mechanism of KtrAB and TrkHA. In KtrB from *Vibrio alginolyticus*, cell-based studies showed that truncations introduced in the intramembrane loop enhance the maximum uptake velocity for K^+^ [21]; and an electron paramagnetic resonance study showed a K^+^-dependent relative motion of the intramembrane loop [22]. In TrkH, mutation of the conserved arginine to alanine enhanced K^+^ flux in a liposome-based assay [20] and electrophysiological recordings with the TrkHA complex showed that truncation of the intramembrane loop changes the response to the ligand and increases the open probability in the absence of ligand [18]. These results, together with the structures of KtrAB and TrkHA, have led to the proposal that the intramembrane loop and the conserved arginine function as a pore gate.

It has been shown that some orthologs of KtrAB are regulated by cyclic-diAMP [23–25], and additionally, in *B*. *subtilis*, the KtrAB operon responds to cyclic-diAMP [26]. Importantly, it is generally accepted that activation of KtrAB and TrkHA involves ATP binding to the KtrA and TrkA gating rings, respectively. Structures of isolated KtrA and TrkA rings show ligand-dependent conformational changes. In TrkHA, the open probability increases with ATP and decreases with ADP [18]. In KtrAB, ion flux is stimulated by ATP and not by ADP; the ADP-bound KtrAB is in a low-activity state, displaying basal activity that is also present in KtrB alone [5,8]. Not much more is understood about the molecular mechanism of activation of these transporters; in particular, the conformational changes induced by ATP and ADP in the KtrAB or TrkHA complexes have not been described.

We present a structure of KtrAB in the low-activity, ADP-bound state and reveal conformational changes in the gating ring and the membrane protein relative to the high-activity ATP-bound KtrAB. We also perform a functional and biochemical characterization of the mechanism of activation. Based on these results, we propose an activation mechanism of the KtrAB complex by ATP.

## Results

### The Structure of the KtrA_ΔC_B Complex

**Fig 1:**
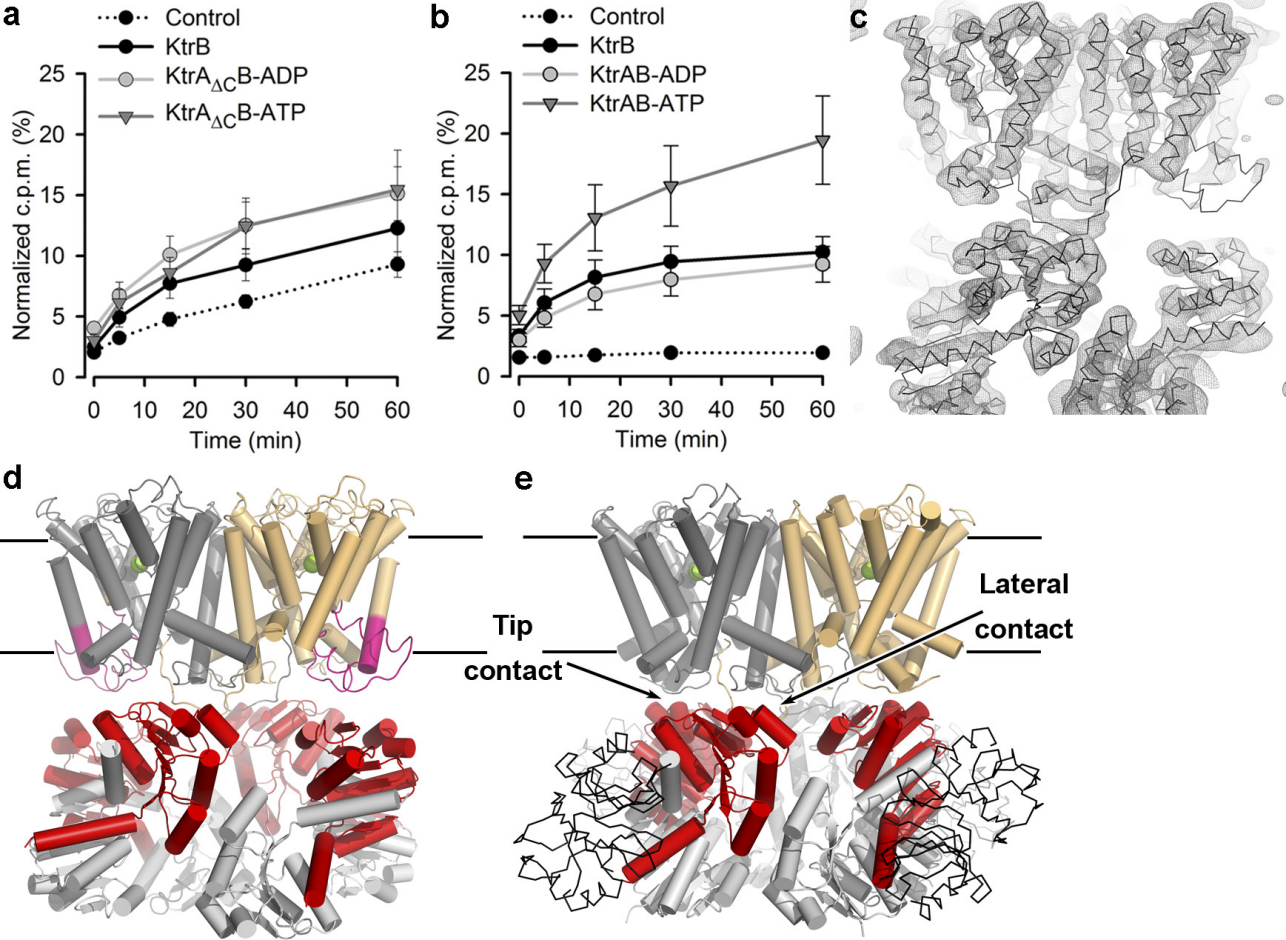
Structure of KtrA_ΔC_B. ^86^Rb^+^ uptake into proteoliposomes with **a**) KtrA_ΔC_B (with ATP or ADP) and KtrB alone (with ATP), or with **b**) wild type KtrAB (with ATP or ADP) and KtrB alone (with ATP). Control liposomes were formed in the presence of KtrA_ΔC_ or KtrA alone; liposome float-up experiments are shown in S3 Fig; KtrA_ΔC_ increases liposome leakiness as reflected in the increased control signal (see raw data in S1 Table). Mean ± standard error of the mean (SEM) values calculated from 5–8 assays from 3 separate liposome preparations. counts per minute (cpm). **c**) Averaged electron-density map of KtrA_ΔC_B in mesh with superposed Cα trace of refined KtrA_ΔC_B structure. Side views of **d**) KtrA_ΔC_B-ADP and **e**) wild type KtrAB-ATP. KtrB dimer is shown at the top and colored dark-grey and wheat, with D1-D2 domain of KtrA_ΔC_B in magenta. RCK rings are shown in light grey and red with N-terminal domain as cartoon and C-terminal domain in KtrAB-ATP as thin Cα trace; K^+^ ions as green spheres; tip and lateral contacts are labeled; putative membrane limits are indicated by horizontal lines. Numerical values of data displayed in panels a and b are included in S1 Data.

To gain structural insights into the mechanism of activation of the KtrAB K^+^ transporter, we crystallized the ADP-bound KtrAB complex from *B*. *subtilis* but could only measure diffraction data to ~8 Å from these crystals. As an alternative, we turned to the KtrA_ΔC_B complex, which is formed by wild-type KtrB and a C-terminal domain truncated form of KtrA (KtrA_ΔC_). This KtrA form lacks residues 145 to 222 that correspond to the whole C-terminal domain (S1E Fig). Ligand-binding studies show that KtrA_ΔC_ from *B*. *subtilis* is able to bind ATP and ADP and crystal structures have shown that it forms an octameric ring [11]. Also, it has been reported that versions of KtrA_ΔC_B from *V*. *alginolyticus* are inactive [8]. We verified by size-exclusion chromatography that the complex between KtrB and KtrA_ΔC_ is formed both in the presence of ADP and ATP (S2A Fig). We also measured KtrA_ΔC_B-mediated ^86^Rb^+^ flux using a liposome-based assay (Fig 1A) and compared it with an equivalent characterization of wild type KtrAB (Fig 1B). As demonstrated before [5], ATP stimulates wild type KtrAB flux relative to ADP and to KtrB alone. Despite a larger background signal (a detailed explanation for this observation can be found in Material and Methods), it is clear that after 1 h, KtrA_ΔC_B-mediated ^86^Rb^+^ uptake levels are identical for KtrA_ΔC_B-ATP and KtrA_ΔC_B-ADP and similar to uptake levels of KtrB alone. Thus, the stimulatory effect of ATP is abolished in KtrA_ΔC_B, leading us to conclude that this complex is trapped in a low-activity state.

We crystallized KtrA_ΔC_B bound to ADP, collected diffraction data to 6 Å (S2 Table), and solved the structure (Protein Data Bank accession number 5BUT) by molecular replacement using the KtrB dimer structure from KtrAB and one of the octameric ring structures previously determined for KtrA_ΔC_ [11]. As in the KtrAB-ATP and TrkHA structures, the asymmetric unit contains two membrane–protein dimers associated with opposing faces of an octameric RCK ring [5,18] (S4 Fig). The calculated electron-density map was then 4-fold and 8-fold averaged in the molecular envelopes corresponding to KtrB and KtrA, respectively. The resulting 6Å electron-density map is of very high quality (Fig 1C), clearly showing the position of all transmembrane and pore helices in KtrB as well as the position of different secondary-structure elements in the KtrA subunits. The final KtrA_ΔC_B-ADP model (Fig 1D and S4 Fig) was generated by manual adjustment of the M1 helix in the KtrB D2 repeat to better fit the map (more details below) and Deformable Elastic Network (DEN) refinement (S2 Table). The final model fits very well in the averaged electron-density map (Fig 1C).

Importantly, we could establish that KtrA_ΔC_B-ADP and KtrAB-ADP are structurally similar and distinct from KtrAB-ATP. Using as search models a collection of different KtrA_ΔC_ ring structures (S5 Fig) and two KtrB structures (from KtrAB-ATP and KtrA_ΔC_B-ADP; Fig 1D and 1E), we performed molecular replacement searches against an 8 Å diffraction dataset collected from wild-type KtrAB-ADP crystals. The six different RCK ring structures together with the two different KtrB structures cover a range of potential conformations of the KtrAB complex and allowed us to get a low resolution model of the KtrAB-ADP structure. The procedure involved sequential searches with the membrane proteins and with the gating rings; for many of these pairs, we were able to obtain molecular replacement solutions that display the expected packing between the membrane protein dimer and the gating ring. The values for the log-of-likelihood (LLG) function (a measure of how well the structural model agrees with the data) are shown in S3 Table; they clearly show that the pair formed by the KtrB homodimer from the KtrA_ΔC_B-ADP together with the KtrA_ΔC_ ring also from the KtrA_ΔC_B-ADP (model 7) has the highest LLG value (LLG = 515). A detailed analysis of the significance and sensitivity of LLG values is presented with S3 Table.

Overall, these LLG results (S3 Table) show that the best fit for the KtrAB-ADP diffraction data is obtained with the protein components of the KtrA_ΔC_B-ADP structure, strongly indicating that KtrA_ΔC_B-ADP is structurally similar to KtrAB-ADP. In contrast, the pair formed by the KtrB homodimer from KtrAB-ATP and KtrA-ATP (without the C-terminal domain) has a LLG = 223 and incorrect packing, indicating that the ATP-bound complex is structurally distinct from KtrAB-ADP. We also determined an averaged density map for the KtrAB-ADP structure using the starting phases calculated from the KtrA_ΔC_B model after rigid body refinement (S6 Fig). Map quality is relatively poor due to the low resolution of the data and limited averaging power, and caution must be exerted during interpretation. In any case, the density supports our conclusion that the KtrA_ΔC_B-ADP and KtrAB-ADP structures are similar, with density that matches the conformation of the KtrA_ΔC_ ring and new density that appears to correspond to the C-terminal domains of full-length KtrA.

**Fig 2:**
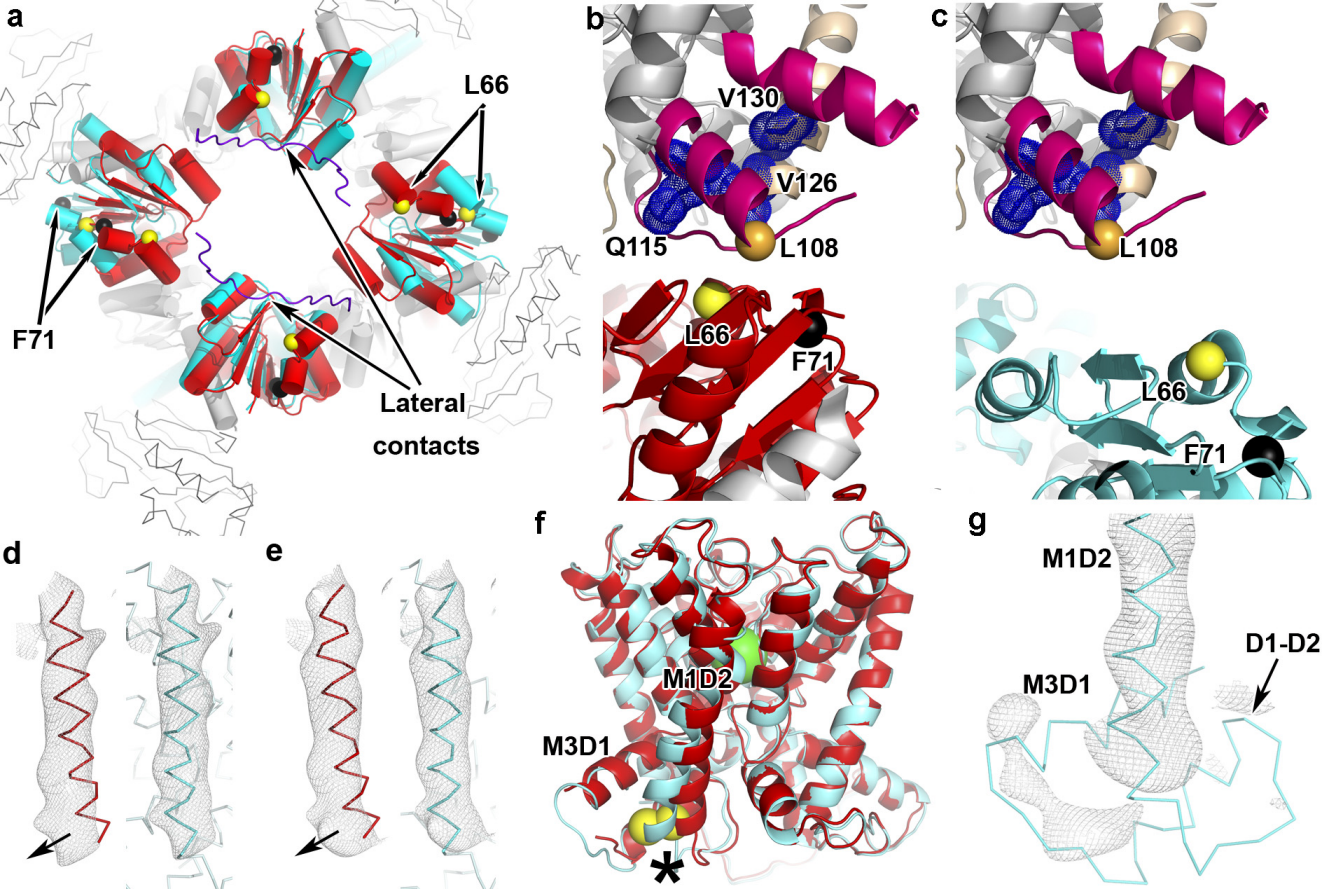
Comparison of KtrA_ΔC_B-ADP and KtrAB-ATP. **a**) Face view of RCK rings from KtrA_ΔC_B-ADP (cyan) and KtrAB-ATP (red) superposed through KtrB homodimers; lateral contacts indicated with KtrB C terminus in purple coil. After superposition of KtrA_ΔC_B-ADP and KtrAB-ATP, a D1-D2 domain from KtrAB-ATP (colored magenta and wheat) is shown together with a KtrA subunit from **b)** KtrAB-ATP (red and grey) and **c)** KtrA_ΔC_B-ADP (cyan). Q115, V126, and V130 are shown in dark blue dot representation; Cα atoms of L66, F71, and L108 shown as spheres. **d)** and **e)** Averaged density (mesh) of M1D2 helix in two different KtrB subunits. KtrAB-ATP M1D2 helices are shown in red (on the left), and KtrA_ΔC_B-ADP M1D2 helices are in cyan (on the right). Arrow indicates shift applied to cytosolic end of helix to bring into density. **f)** Overall view of superposed KtrB subunits from KtrA_ΔC_B-ADP (cyan) and KtrAB-ATP (red). Star indicates moved M1D2 helix; I127 Cα in the two structures is shown as yellow sphere; K^+^ ion as green sphere. **g)** Averaged density (mesh) of D1-D2 domain region together with refined KtrA_ΔC_B-ADP model. Lack of density for M3D1 and D1-D2 helices is apparent.

The overall organization of KtrA_ΔC_B-ADP and KtrAB-ATP, including the relative disposition of the KtrB homodimer subunits, is similar (Fig 1D–1E). Importantly, the KtrA_ΔC_ ring is asymmetrically expanded along the diagonal defined by the tip contacts (Fig 2A), revealing that in the KtrAB complex the ligand-dependent conformational change of the RCK ring occurs along the tip contacts. As a consequence of this change, the KtrB-KtrA interface contact sites are affected differently. In the lateral contacts, the spatial relationship between the KtrB C termini and two KtrA subunits is almost unaltered. The Cα-Cα distance separating F71 (or L66) in the two lateral contact KtrA subunits is almost unchanged: ~66 and ~65 Å for F71, ~48 and ~49 Å for L66, in KtrAB-ATP and KtrA_ΔC_B-ADP respectively. In the tip contact KtrA subunits, the Cα-Cα distance F71 distance increases from ~68 Å (KtrAB-ATP) to ~87 Å (KtrA_ΔC_B-ADP), and for L66, from ~50 to ~76 Å. This expansion also includes a KtrA movement away from the membrane protein (Fig 2B and 2C); the distance separating a reference spatial position (the Cα atom of KtrB-L108 in KtrAB-ATP) from the Cα of KtrA-F71 increases from ~9 Å in KtrAB-ATP to ~21 Å in KtrA_ΔC_B-ADP.

Although the overall conformations of the KtrA_ΔC_ ring and isolated KtrA-ADP ring [5] resemble each other, with one diagonal of the ring longer than the other, they are different structures (S7A Fig). This is clearly seen in the Cα-Cα distances between F71 residues in opposing ring subunits. In the isolated KtrA-ADP structure, these distances are ~68 and ~82 Å, while in KtrA_ΔC_ from KtrA_ΔC_B-ADP the distances are ~66 and ~87 Å. The intradimer arrangement in KtrA_ΔC_B-ADP is also different from the one seen in the isolated full-length KtrA-ADP structure (S7B Fig); the angle between the KtrA dimer subunits in KtrA_ΔC_B-ADP has changed by ~15° relative to KtrA-ADP. In any case, the angle difference between KtrA_ΔC_ and KtrA in KtrAB-ATP is even larger, being close to 30° (S7C Fig).

Importantly, the averaged electron-density map also reveals structural changes in the KtrB protein. As in the KtrA ring, all apparent changes are centered on the KtrB tip contact region, and no alterations are detected around the lateral contact. The position of the cytosolic end of the M1 transmembrane helix in repeat D2 (M1D2) in the averaged density is clearly different from the one seen in the KtrAB-ATP structure (Fig 2D and 2E and S8 Fig); the need for a positional adjustment of the helix is also detectable in a difference map (S9 Fig). The M1D2 helix was adjusted with an outward movement of the cytosolic end so that the Cα atom of I127 is shifted by ~3–4 Å (Fig 2D–2F). The better fit of the final model to the averaged electron-density map (Fig 2D–2E and S8 Fig) is supported by an increase in the map correlation coefficient calculated for the main chain and Cβ atoms of the cytosolic half of the M1D2 helices (residues 128 to 140) in the 4 KtrB asymmetric unit copies: 0.50 in KtrAB-ATP and 0.71 in KtrA_ΔC_B-ADP. As a result of this conformational change, the M1D2 helix in KtrA_ΔC_B-ADP has been straightened while in KtrAB-ATP it is bent towards the cytosolic pore. In addition, while all other KtrB helices are visible in the averaged electron-density map (including the pore helices and the M3 helices of repeats D2, D3, and D4), there is no density for the two helices just before M1D2, M3D1, and the D1-D2 loop helix (Fig 2G); in KtrAB-ATP, these two helices, together with M1D2, form a domain-like structure (the D1-D2 domain) that functions as a KtrB foot on the tip contact (Fig 1D and 1E). The lack of density for the M3D1 and D1-D2 helices in the 6Å resolution KtrA_ΔC_B-ADP averaged map probably results from either unwinding of the helices, as a manifestation of increased local disorder, or from a breakdown of the KtrB “homodimer” symmetry in this region.

### Remodeling of the Tip-Contact Region during Activation

To explore in more detail the ligand-dependent remodeling of the D1-D2 domain in KtrB, we evaluated the ligand-dependent accessibility of cysteine residues introduced in the D1-D2 domain. We engineered single-cysteine mutations in a cysteineless KtrB (Fig 2B and 2C): Q115C is a semiburied residue on the D1-D2 loop; V126C and V130C are on the M1D2 helix and are semi- (V126C) or fully buried (V130C). These mutants were assembled with cysteineless KtrA (KtrA_C0_), and we confirmed that the properties of the mutant complexes are similar to wild type KtrAB (S10 Fig): by size-exclusion chromatography, we verified that the complexes are assembled in the presence of ATP and ADP and, using the ^86^Rb^+^ flux assay, we verified that the mechanism of ligand activation (stimulation by ATP relative to ADP) is not markedly altered. Using a fast injection system, we mixed the complex with ~20-fold molar excess DTNB (5,5’-dithio-bis(2-nitrobenzoic acid)) in the presence of ATP or ADP and followed the reaction time course. DTNB, or Ellman’s reagent, reacts rapidly with reduced thiols in tissues and proteins [27–29] and irreversibly in our experimental conditions, generating stoichiometric amounts of the yellow thio-nitrobenzoate (TNB), which absorbs at 412 nm.

**Fig 3:**
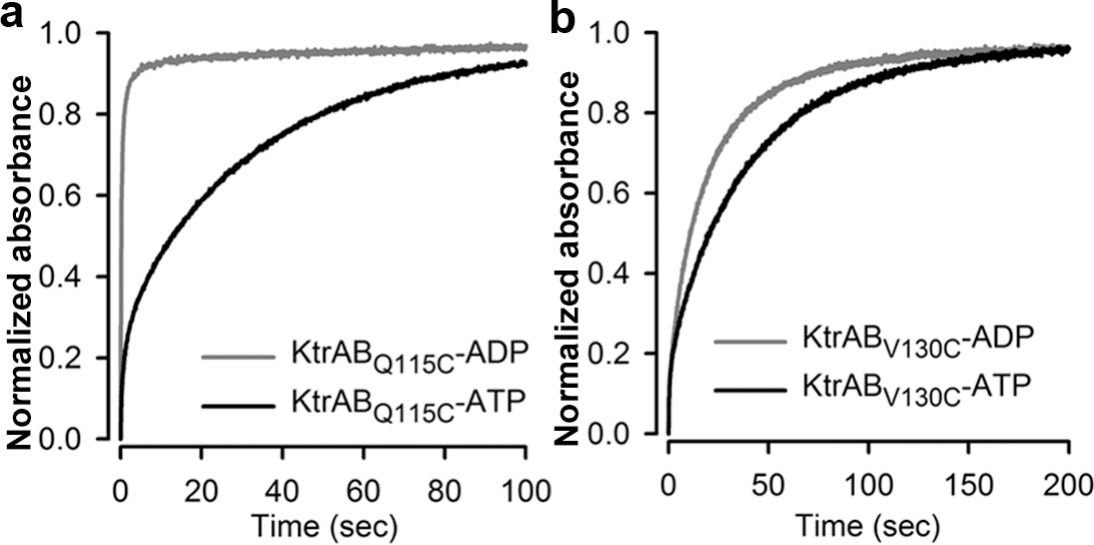
Remodeling of the tip contact. Time course of DTNB modification reaction for **a)** KtrB_Q115C_ and **b)** KtrB_V130C_ in complex with KtrA_C0_ bound to ADP (gray) or ATP (black). Representatives of four separate modification reactions are shown, normalized to maximum (last recorded) value. For each mutant, the final absorption values between ADP and ATP varied by less than 30%, showing that differences in initial cysteine oxidation levels are small. The initial fast jump observed in the time course is due to the time resolution of our system (100 ms). Numerical values are included in S1 Data.

The DTNB modification time courses for Q115C (Fig 3A) clearly show that the reaction is much faster (~65 times faster) in the presence of ADP than in the presence of ATP; the modification halftimes are ~0.2 sec and ~13 sec with ADP and ATP, respectively (Table 1). With the KtrAB_V126C_ mutant, the reactions are remarkably fast in the presence of either ligand, and we cannot detect a difference in reactivity (Table 1, S10B Fig), but the modification halftime for KtrAB_V130C_ (~10 s) is also shorter for ADP than with ATP (~21 s) (Fig 3B and Table 1). Faster reaction time courses for ADP relative to ATP for two different cysteine positions in the D1-D2 domain are consistent with an increase in cysteine accessibility to DTNB in the ADP-bound state and support the proposal that the domain has undergone a structural change. This biochemical analysis together with our structural comparison establishes that during ligand activation the RCK ring conformational change is associated with remodeling of the tip contact interface and D1-D2 domain.

**Table 1:** DTNB modification reaction halftimes for mutant cysteines.

	ADP (s)	ATP (s)
**Cysteines introduced in the D1-D2 domain**		
Q115C	0.23 ± 0.01	12.75 ± 0.48
V126C	0.21 ± 0.01	0.21 ± 0.02
V130C	10.48 ± 0.09	20.95 ± 0.39
**Cysteines introduced in the cytosolic pore**		
N119C	0.24 ± 0.01	0.55 ± 0.01
P121C	0.23 ± 0.02	0.91 ± 0.05
F443C	0.48 ± 0.01	63.73 ± 0.43
T444C	52.43± 4.02	386.55 ± 106.99 *

SEM values are determined from 3–4 separate modification reactions.

* Halftime for T444C with ATP is underestimated since the reaction is very slow and does not reach its end point.

### The KtrB Cytosolic Pore Becomes Narrower during Activation

**Fig 4:**
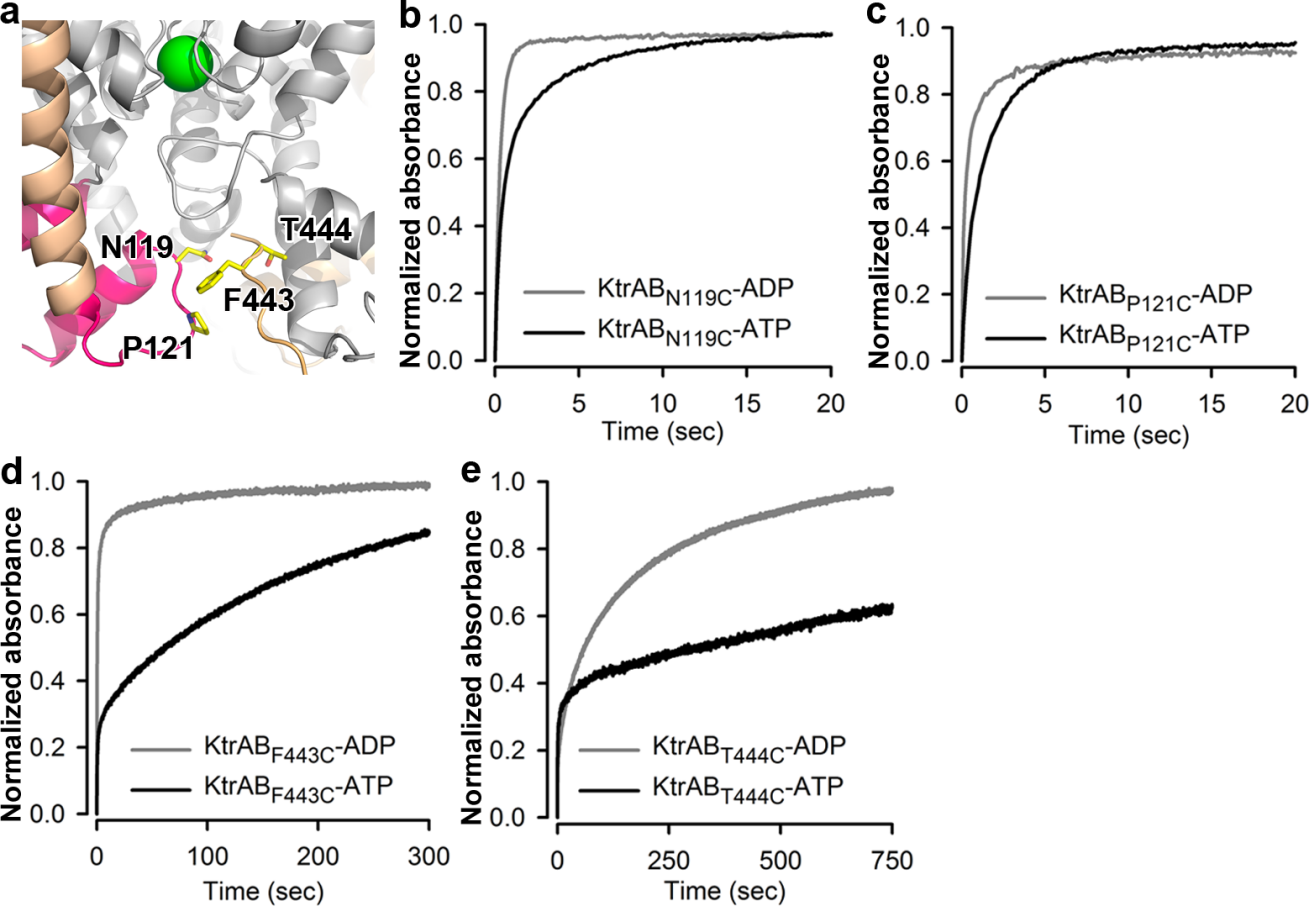
Narrowing of cytosolic pore. **a)** View of cytosolic pore wall residues (in yellow stick) that were mutated to cysteines. KtrB C-terminus shown as orange coil; D1-D2 domain from KtrAB-ATP shown in magenta and wheat; K^+^ ion as green sphere. Time course of DTNB modification reaction for **b)** KtrB_N119C_, **c)** KtrB_P121C_, **d)** KtrB_F443C_ and **e)** KtrB_T444C_ in complex with KtrA_C0_ bound to ADP (gray) or ATP (black). Representatives of four separate modification reactions are shown. Normalized to maximum (last recorded) value. The initial fast jump in the time course (very noticeable in ATP time courses of F443C and T444C) is due to the time resolution of our system (100 ms). For N119C, P121C, and F443C, the final absorption values between ADP and ATP varied by less than 30%, showing that differences in cysteine oxidation are small; this value could not be determined for T444C with ATP due to its very slow reactivity. Numerical values of data displayed in panels b–e are included in S1 Data.

A central feature of the activation mechanism of many K^+^ channels is the opening of a cytoplasmic gate and increased access to the cytosolic pore. Since Trk/Ktr/HKT proteins are structurally similar to K^+^ channels, we asked whether the same happens in KtrAB during activation. To assess the ligand-dependent conformational changes in the pore of KtrB, we made use of the DTNB assay (introduced above) and probed ligand-induced changes in accessibility of cysteine residues introduced on the wall of the cytosolic pore of KtrB. In the KtrAB-ATP structure, N119C is positioned at the mouth of the pore, while P121C, F443C, and T444C are positioned deep in the pore (Fig 4A). These mutants showed no alteration in their ability to assemble with KtrA and retained the ATP stimulation effect, although reduced for F443C (S11 Fig).

The DTNB modification halftimes measured for the four cysteine mutants were consistently shorter with ADP than with ATP (Fig 4B–4E, Table 1), showing that all cysteine thiol groups are less reactive in the ATP-bound state. This trend across four different positions strongly indicates a reduction in DTNB accessibility. These results, together with the ligand-dependent repositioning of the M1D2 helix towards the cytosolic pore in KtrAB-ATP (Fig 2F), suggest that the cytosolic pore of KtrAB becomes narrower upon ATP activation.

### Functional and Biochemical Characterization of the Intramembrane Gate

**Fig 5:**
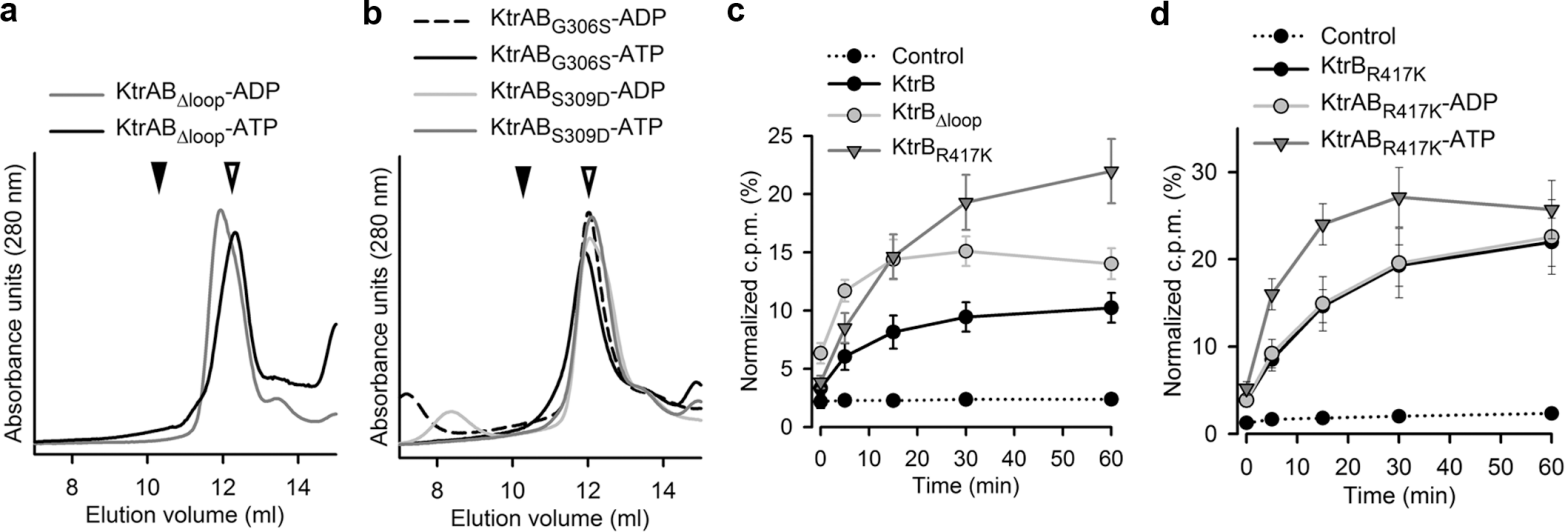
Properties of intramembrane gate mutants. Size-exclusion profiles of intramembrane loop **a)** truncation and **b)** G306S and S309D single mutants assembled with KtrA in the presence of ATP and ADP. Inverted arrow heads indicate elution volumes of KtrAB complex (closed symbol) and of separate components (open symbol). SDS-PAGE of fractions are shown in S12 Fig; **c)** Time course of ^86^Rb^+^ uptake for KtrB wt and mutants (KtrB_Δloop_, KtrB_R417K_) in the absence of KtrA. **d)** Time course of ^86^Rb^+^ uptake for KtrB_R417K_ alone and KtrAB_R417K_ with ADP or ATP. Liposome float-up experiments are shown in S13 Fig. Numerical values of data displayed in panels c–d are included in S1 Data.

The intramembrane loop and the conserved arginine are thought to function as a gate in KtrAB and TrkHA. We analyzed the functional impact of mutations both in the loop and in the arginine (R417). We truncated the intramembrane loop (KtrB_Δloop_—truncation of residues G306 to A311), mutated two residues in the loop (KtrB_G306S_ and KtrB_S309D_), and mutated the conserved arginine to a lysine (KtrB_R417K_). Strikingly, the intramembrane loop seems to be very sensitive to alterations since both the truncation (Fig 5A) and the two single point mutations (Fig 5B) have a destabilizing effect on the interaction between KtrB and KtrA. Hanelt and colleagues [21] described the same effect for truncations of the intramembrane loop in *V*. *alginolyticus* KtrB but did not observe destabilization with a mutation equivalent to G306S. In contrast, KtrB_R417K_ assembles with KtrA (S2B Fig) in the presence of ADP or ATP.

Flux assays with liposome-reconstituted KtrB_Δloop_ and KtrB_R417K_ show that the uptake in KtrB_Δloop_ is faster than in the KtrB wild type protein (single exponential time constants [τ] are ~5 min for KtrB_Δloop_ and ~11 min for wild type KtrB) as expected if an obstacle to ion flow has been removed (Fig 5C). On the other hand, the rate of uptake in KtrB_R417K_ alone (τ ~22 min) is slow. More interestingly, the uptake rate of KtrAB_R417K_-ATP (τ ~6 min) is as fast as in KtrB_Δloop_ (Fig 5D) while KtrAB_R417K_-ADP (τ ~22 min) is comparable to wild type KtrAB (τ ~19 and ~17 min, for KtrAB-ATP and KtrAB-ADP respectively). This suggests that the conservative substitution in the KtrAB_R417K_ complex is functionally similar to the removal of the intramembrane loop from the cytosolic pore of KtrB. Note however that this effect is ligand-dependent since it only occurs when KtrB_R417K_ is associated with KtrA with bound ATP and not with ADP.

Overall, changes in the intramembrane loop or in R417 modify the ion permeation properties in a way that is consistent with a role in the intramembrane gate.

## Discussion

The structures of the isolated RCK rings from the KtrAB and TrkHA ion transporters led to the proposal that ligand-induced conformational changes in the RCK rings (KtrA or TrkA) are at the basis of the activation mechanisms of the Ktr and Trk ion transporters [5,8,18]. In KtrAB in particular, it was shown that the isolated KtrA ring expands asymmetrically upon exchange of ATP for ADP. However, those studies did not show how the asymmetric expansion/contraction occurs within the complex. In fact, no conformational changes had been demonstrated in the KtrAB and TrkHA complexes.

**Fig 6:**
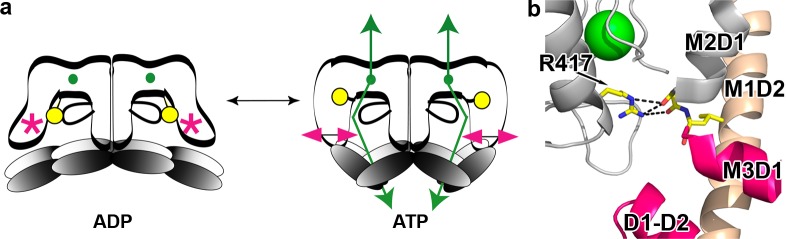
Proposed mechanism of activation in KtrAB. **a)** Model for KtrAB ligand activation: ion flux stimulation by ATP (green arrows) results from a change in the conformation of the RCK ring (gray ovals) and D1-D2 domain (magenta arrows). These changes alter the interactions established by R417 (yellow circle) with the D1-D2 domain (magenta star). **b)** View of R417 side chain in KtrAB-ATP structure establishing contacts with main chain carbonyls in the C-terminus of M2D1 helix. K^+^ ion as green sphere; helices are labeled.

We have now presented structural, biochemical, and functional evidence establishing that KtrAB activation by ATP involves a ligand-dependent asymmetrical contraction of the KtrA gating ring, changes in the tip contact interface and remodeling of the D1-D2 domain (Fig 6A).

Our results also reinforce the notion that the intramembrane loop and the conserved arginine (R417) form the gate in KtrAB and TrkHA [18,20–22,30] (see S1C Fig). Destabilization of the interaction between KtrB and KtrA that results from intramembrane loop modifications appears to be more consistent with the loop having a structural role than being a mobile component of a gate. Our work brings forth the functional importance of R417 in the activation mechanism; the conservative mutation R417K in the KtrAB_R417K_ complex is functionally similar to removing six residues from the intramembrane loop, resulting in an increased rate of ion flux; strikingly, this functional effect is ligand-dependent, since in KtrAB_R417K_ the increase in the rate of flux is only observed with ATP. The importance of R417 in the activation mechanism is further reinforced by inspection of the KtrAB-ATP structure. This residue interacts with the main chain carbonyls at the C-terminal end of the M2D1 helix (Fig 6B), a helix that is directly connected to and abuts the D1-D2 domain. It is thus possible to envisage that contraction/expansion of the KtrA ring and rearrangements of the tip contact region and D1-D2 domain will alter the interactions between R417 and the C-terminal end of the M2D1 helix, affecting therefore the intramembrane gate (Fig 6A).

Block of the ion permeation pathway by the intramembrane gate in the KtrAB-ATP structure [5] remains a puzzle. One possibility is that in detergent, the ion transporter is not able to adopt the fully activated conformation. Another possibility is that ATP binding is connected to several different conformations, and in the KtrAB-ATP structure, we captured a conformation where the intramembrane gate blocks the pathway, not unlike the desensitized state of some ligand-gated ion channels [31–34].

A remarkable aspect of the ligand-dependent mechanism of activation in KtrAB that has been revealed by our data is that the KtrA ring (an RCK ring) contracts, and the KtrB cytosolic pore narrows down with activation. This is very different from what happens in the related MthK or BK potassium channels that are also regulated by ligand binding to their RCK rings. In these channels, binding of the activating ligand (Ca^2+^) to the RCK ring results in an expansion of the ring and widening of the cytosolic pore [15,35,36]. Moreover, activation in the majority of K^+^ channels results in increased accessibility to the cytosolic ion pore [37–40]. An explanation for this apparently contradictory result is found in the KtrAB-ATP structure. It shows that, despite narrowing of the pore, there is a water-accessible conduit up to the intramembrane gate. It is therefore likely that narrowing of the cytosolic pore during activation results from the reorganization of a long-range allosteric network that has to connect the tip contact (and the RCK ring) and the intramembrane gate. Upon ATP activation, the cytosolic pore narrows down but remains wide enough to allow easy permeation of fully or partially solvated potassium ions.

In summary, despite the structural similarity to K^+^ channels, the mechanisms of ligand activation in Trk/Ktr/HKT ion transporters are unique.

## Materials and Methods

### Protein Expression and Purification

N-terminal His-tagged KtrB (GenBank: KIX81591.1) cloned in a modified pET-24d vector (Novagen) was overexpressed in *Escherichia coli* BL21(DE3). Cells grown in LB media at 37°C were induced with 0.5 mM IPTG for 2.5 h. Cells were lysed in protease inhibitor-supplemented buffer (50 mM Tris-HCl pH 8.0, 120 mM NaCl, 30 mM KCl) and KtrB extracted with 40 mM DDM (*n*-dodecyl-β-D-maltoside, solgrade from Anatrace) overnight at 4°C. Spin-cleared lysate was loaded into a Talon metal affinity resin (Clontech) and washed with 50 mM Tris-HCl pH 8.0, 270 mM NaCl, 30 mM KCl, 1 mM DDM followed by buffer A (50 mM Tris-HCl pH 8.0, 120 mM NaCl, 30 mM KCl, 1 mM DDM) supplemented with 20 mM imidazole. Protein was eluted with buffer A supplemented with 150 mM imidazole and immediately 3-fold diluted in buffer A supplemented with 15 mM DTT and 1.5 mM TCEP. Eluted fractions were concentrated to ~2.5 mg/ml and incubated overnight at 4°C with thrombin for tag cleavage. At this point, the protein was either assembled with KtrA (for protein crystallography) or further purified by size exclusion with a Superdex-200 column.

Wild type KtrA (GenBank: KIX81590.1) and cysteine-less KtrA (KtrA_C0_—C22V, C178V, C109S, C204S) were overexpressed in *E*. *coli* BL21(DE3), grown in LB media at 20°C, after ~14–16 h induction with IPTG. Cells were lysed in 50 mM Tris-HCl pH 7.5, 50 mM KCl, 5 mM DTT, and the spin-cleared lysate was loaded into an anion exchange column. KCl gradient-eluted fractions containing KtrA were pooled and incubated with ADP-agarose resin (Innova Biosciences) overnight at 4°C. Beads were washed thoroughly with Buffer B (50 mM Tris-HCl pH 8.0, 150 mM KCl, 1 mM TCEP), and protein was eluted in buffer B supplemented with either 5 mM ADP or ATP (sodium salts). Protein was concentrated to ~3 mg/ml and further purified by size exclusion with a Superdex-S200 column using buffer C (50 mM Tris-HCl pH 7.5, 150 mM KCl, 5 mM DTT). KtrA in solution was supplemented with 1 mM adenine-nucleotide from a 100 mM stock in 1 M Tris-HCl pH 7.5.

The KtrA_ΔC_ construct includes two N-terminal KtrA domains in tandem (residues 1–144 + 7–144), connected by a linker (-LEGS-), and cloned in a modified pET-24d vector (Novagen). This tandem protein does not aggregate as easily as a single N-terminal KtrA domain. Purification of KtrA_ΔC_ followed the procedure described before [11]. Briefly, the protein was overexpressed in BL21(DE3) by overnight induction at 20°C; cell lysate in buffer D (50 mM Tris-HCl pH 8.5, 120 mM NaCl, 30m M KCl) was loaded into a His-tag affinity column, and protein was eluted with buffer supplemented with 150 mM Imidazole; after adding thrombin for cleavage of tag, protein was dialysed overnight at 4°C against buffer D supplemented with 5 mM DTT; protein was concentrated and further purified in a size-exclusion Superdex-200 column with 20 mM Tris pH 8.5, 150 mM NaCl, 5 mM DTT before assembling with KtrB.

For protein crystallography, the KtrA_ΔC_B-ADP and KtrAB-ADP were prepared by mixing purified KtrB with an excess of pure KtrA_ΔC_ or KtrA. The mixture was concentrated and purified in a size-exclusion Superdex-200 column with 20 mM Tris-HCl pH 8.0, 120 mM NaCl, 30 mM KCl, 5 mM DTT, 1.5 mM 6-cyclohexyl-1-hexyl-β-D-maltoside (cymal-6, anagrade, from Anatrace). Collected fractions were concentrated to 7–10 mg/ml after adding ADP to 1 mM.

For biochemical experiments, size-exclusion purified KtrB (wild type or cysteine mutants) was mixed with an excess of KtrA (wild type, KtrA_C0_ or KtrA_ΔC_) and directly used in the assays. For DTNB modification assays, KtrA_C0_ was prepared in the absence of DTT and TCEP throughout the complete purification procedure to avoid cross reaction of DTNB with the reducing agent.

### Crystallization

KtrA_ΔC_B was crystallized by mixing 1 μl of protein complex at ~10 mg/ml with 1 μl of crystallization solution (100 mM Tris-HCl pH 8.5, 18%–22% PEG400, 100–200 mM CaCl_2_) and incubating at 20°C. Crystals were cryoprotected by increasing PEG400 to 29% by adding directly to drop from 45% stock, equilibrating for 2 d, and then mixing cryosolution (45% PEG400, 250 mM CaCl_2_, 15 mM cymal-6, 100 mM Tris-HCl 8.5) before dropping crystal into liquid nitrogen. KtrAB-ADP was crystallized by mixing 1 μl of protein complex at ~7 mg/ml with 1 μl of crystallization solution (100 mM Hepes pH 7.5, 1 M NaCl, 10% PEG4000) and incubating at 20°C. Crystals were cryoprotected by transferring sequentially to 100 mM Hepes pH 7.5, 25% PEG4000, 1 M NaCl, 1.5 mM cymal-6, 0.5 mM ADP with 10% glycerol, and to the same solution with 25% glycerol, before dropping crystal into liquid nitrogen.

### Data Processing and Structure Determination

Diffraction data from KtrA_ΔC_B-ADP crystals were collected at Soleil Proxima 2 and from KtrAB-ADP at ESRF ID14-4 and processed with XDS [41]. KtrA_ΔC_B-ADP structure was solved by molecular replacement with PHASER (CCP4 package [42]) using the KtrB dimer from KtrAB-ATP (PDB 4J7C) and the KtrA octameric ring (without C-terminal domain) (PDB 2HMS). 4-fold (around KtrB) and 8-fold (around KtrA_ΔC_) averaging was performed using DMmulti (CCP4 package [42]) with masks calculated with MAMA. Rigid body refinement of KtrA_ΔC_B was performed with PHENIX [43] using a single data bin and 12 groups (1 for each of the KtrA_ΔC_ and KtrB subunits). DEN-refinement [44] was performed using SBGrid Science Portal server (https://portal.sbgrid.org/d/apps/den) using a single overall B-factor (no group or individual B-factor refinement), restrained NCS (two groups, corresponding to KtrA_ΔC_ and KtrB, and restraining weight of 300), no positional refinement, DEN restraints with 3–15 Å cutoff, and no sequence or chain separation limits. Combinations of starting annealing temperatures, W_DEN_ and γ factor were tested, and the best solution, as a combination of Ramachandran plot and R_free_, was obtained with annealing temperature of 3,000 K, W_DEN_ = 300 and γ = 1.0. Lowest R_free_ solution differed by 0.23% in R_free_ from selected coordinates but presented much worse stereochemistry (only 67% of residues in the favored region of a Ramachandran plot).

### Preparation of Proteoliposomes

Proteoliposomes were prepared as previously described [5] with modifications. Polar *E*. *coli* lipids (Avanti) in chloroform were dried under a stream of argon. Residual solvent was removed by pentane wash and redrying of the film. Lipids were resuspended at 10 mg/ml in swelling buffer (150 mM KCl, 10 mM Hepes, 5 mM N-methylglucamine, pH 7.4) using a bath sonicator. Lipids were solubilized by adding 40 mM decylmaltoside (solgrade from Anatrace) from powder and left for ~2 h with gentle agitation at room temperature. KtrAB complex was prepared and dialyzed overnight at 4°C prior against 150 mM KCl, 10 mM Hepes, 5 mM N-methylglucamine, 5 mM DTT, 0.5 mM DDM, pH 7.4, with nucleotide. Adenine nucleotide levels were adjusted during dialysis to spontaneously reach 20 μM after reconstitution (calculated from protein concentration and the corresponding dilution factor [volume added to lipid aliquots]). Protein was added to solubilized lipids at 1:100 (w:w) protein-to-lipid ratio and incubated for 30 min at room temperature. The amount of protein added to 100 μl aliquot of lipids was adjusted to contain 10 μg KtrB both when reconstituted alone or as KtrAB complex. Detergent was removed by adsorbing SM-2 Biobeads (BioRad): the protein–lipid mix was incubated twice with fresh BioBeads at 10:1 (w:w) bead-to-detergent ratio at room temperature for 1 h, followed by an overnight incubation at 4°C at a 20:1 (w:w) bead-to-detergent ratio. Control liposomes were prepared similarly, except that only KtrA (wt, KtrA_C0_ or KtrA_ΔC_) was added to the solubilized lipids.

### ^86^Rb^+^ Flux Assay

Assay was performed as previously described [5] with small modifications. A K^+^ gradient was formed by spinning 100 μl aliquot of liposomes through a 1.5 ml bed of Sephadex G50 (fine) preswollen in sorbitol buffer (20 μM KCl, 150 mM sorbitol, 10 mM Hepes, 5 mM N-methylglucamine, pH 7.4) and supplemented with 20 μM adenine nucleotide. Liposomes were then mixed with twice the volume of ^86^Rb^+^ assay buffer (^86^Rb^+^ at ~2.000 counts/μl in sorbitol buffer) and the reaction was run for 60 min. At selected time points, 100 μl aliquots of uptake reactions were loaded into Dowex cation exchange columns (prewashed in 150 mM sorbitol solution plus 5 mg/ml bovine serum albumin and pre-equilibrated in 150 mM sorbitol solution) and eluted with 6% sorbitol solution. The eluate (liposomes with accumulated ^86^Rb^+^) was collected in 4 ml scintillation vials and mixed with Optiphase scintillation fluid at 1:1 (v:v). At the end of each experiment (at 60 min), valinomycin was added to the last 100 μl aliquot of the uptake reaction at a final concentration of 900 nM, incubated for 6 min and then loaded into Dowex columns as described above. The uptake of ^86^Rb^+^ into liposomes was expressed as the percentage of the counts measured after valinomycin treatment (this step corrects for reconstitution variability among different batches of liposomes).

In detergent, both KtrB and TrkH associate with the respective RCK ring partners as either one membrane–protein dimer or as two dimers, with each dimer binding to opposite faces of the ring (see S4 Fig) [5,18]. To favor the formation of the complex with one KtrB dimer plus one KtrA octamer, we performed the liposome reconstitution with a fixed amount of KtrB and an excess of KtrA or KtrA_ΔC_. Any unforeseen effect of the free KtrA or KtrA_ΔC_ protein fraction on the liposomes was controlled by reconstituting liposomes with KtrA or KtrA_ΔC_ alone. These liposomes are used to evaluate the background signal of the assay; it is however important to realize that the fraction of free regulatory protein in the KtrAB or KtrA_ΔC_B reconstituted liposomes is much lower than in these background liposomes since most of the protein will be present in a membrane protein complex.

The increased background signal observed in the KtrA_ΔC_B flux assay results from a reduction in the radioactivity uptake observed with valinomycin flux in the KtrA_ΔC_ liposomes. Since valinomycin values are used for normalization of the assay, smaller valinomycin values for KtrA_ΔC_ liposomes result in normalized flux uptake that is larger than for full-length KtrA-liposomes. Valinomycin flux reduction is consistent with increased leakiness of the KtrA_ΔC_ liposomes; leakiness in the KtrA_ΔC_ liposomes decreases the electrical potential gradient in these liposomes and lowers the uptake of ^86^Rb^+^. The molecular basis for this effect is not known. As stated in the previous paragraph, leakiness would be more pronounced in the KtrA_ΔC_-liposomes since the amount of free KtrA_ΔC_ protein is higher in control liposomes than in KtrA_ΔC_B liposomes.

### Liposome Float-Up Experiments

50 μl of proteoliposomes in 40% (w/v) sucrose were put into the bottom of polyallomer centrifuge tubes (Beckman Coulter) and sequentially overlaid with 100 μl of 20% sucrose solution and 50 μl of 5% sucrose solution, forming a discontinuous gradient from bottom to top. The samples were centrifuged at 100,000 *g* for 60 min at 4°C in an Airfuge Ultracentrifuge (Beckman Coulter) using the A-100/18 rotor. Fractions of 40 μl each were collected from top to bottom and protein was detected by western blotting.

Samples were run in 10% or 15% polyacrylamide gel, blotted onto nitrocellulose membrane, and probed with anti-KtrB polyclonal antibody (overnight incubation at 4°C) or anti-KtrA polyclonal antibody (2 h at room temperature), respectively. Detection was done by incubation with anti-rabbit IgG conjugated with peroxidase (Sigma) for 30 min at room temperature and using Amersham ECL Prime western blotting detection reagents.

### Cysteine Modification with DTNB

Just prior to initiating the DTNB modification reaction, KtrB in DTNB buffer (20 mM Tris-HCl pH 8.0, 120 mM NaCl, 30 mM KCl, 5 mM EDTA, 0.5 mM DDM) was mixed with excess of KtrA_C0_ in buffer C without DTT while keeping the detergent concentration well above its critical micellar concentration. For each reaction KtrB was present at a concentration of 14–20 μM KtrB. The protein was loaded into one input channel of a rapid mixing device (SFA-20 Rapid Kinetics Accessories, Hi-TECH, TgK Scientific). The second channel was loaded with 400 μM DTNB dissolved in DTNB buffer. Solutions were mixed upon manual injection into the flow cell, which was placed in the cuvette holder of a Shimadzu UV-2401 spectrophotometer. As a result of the 1:1 mixing of protein and reagent, the final concentration of the reactants is halved. DTNB is a water-soluble compound and partitions weakly into detergent micelles, which makes it an ideal candidate for our experiments with a detergent-solubilized membrane protein. The release of TNB from DTNB was followed at 412 nm with 1 nm open slit with 10 points/sec sampling rate. Before each assay, we filled the flow cell with DTNB and blanked the system to eliminate any background signal.

With ~20-fold molar excess of DTNB over protein and in the absence of reducing agents, we were expecting to observe an apparent first order reaction. Instead, we consistently observed multiple components in the modification time course. Control experiments with reduced glutathione and DTNB (both in the presence and absence of 0.5 mM DDM) showed a first-order reaction demonstrating that the multiple components seen with the membrane protein were not the result of our experimental setup or the presence of detergent. Fitting the time course with multiple exponentials revealed that one exponential component dominated with an amplitude of at least 70%. We concluded that the complex reaction kinetics are a feature of protein modification with DTNB that result from the natural existence of population variability. In any case, instead of using a mean time constant obtained from a multiple exponential fit to describe the time course, we used the reaction halftime. When using sufficiently long experimental recording times, the reaction halftime minimizes the error that results from the presence of a very slow component in the time-course.

Modification of accessible thiols with DTNB produces stoichiometric amounts of TNB, and as a consequence the final absorbance value is defined by the initial concentration of reactive cysteine. Differences between the final absorbance values of a particular mutant with ATP and ADP were less than 70%. Note, that after 1 h incubation of DTNB with unfolded KtrAB protein (mixed with 3 M guanidium-hydrochloride) we obtained ~1 cysteine/KtrB subunit for all tested constructs, as expected.

## Supporting Information

S1 Data Numerical values of data displayed in Figs 1A and 1B, 3A and 3B, 4B–4E, 5C and 5D, S10A–S10C, and S11A–S11D. (XLSX)

S1 Fig Similarities between KtrAB and K+ channels. Extracellular views of **a)** KtrB homodimer and **b)** KcsA K^+^ channel. Side views of **c)** a single KtrB subunit without the 2nd repeat and **d)** KcsA pore without a channel subunit. Each repeat and subunit is colored a different shade of blue. KtrB repeats are labeled. Transmembrane and pore helices of one repeat or subunit are labeled. The intramembrane loop and conserved arginine are indicated. K^+^ are shown as green spheres bound in the selectivity filter. Structure of RCK octameric ring in **e)** KtrA (with a C-terminal domain indicated) and in **f)** MthK K^+^ channel. Alternate subunits are shown in red and gray.
(TIF)

S2 Fig Assembly of KtrA_ΔC_B and KtrAB_R417K_. Size-exclusion profiles of **a)** KtrB assembled with KtrA_ΔC_ in the presence of ATP and ADP and **b)** KtrB_R417K_ mutant assembled with KtrA in the presence of ATP and ADP. Closed inverted arrowhead indicates elution volume of KtrAB complex; open arrowhead indicates elution volume of individual components. SDS-PAGE of size-exclusion fractions are shown as insets. Inset in **a)** 1- KtrB preparation before size exclusion; 2- KtrA_ΔC_+KtrB with ADP eluting at closed arrow; 3- KtrA_ΔC_+KtrB with ADP eluting at open arrow; 4- KtrA_ΔC_+KtrB with ATP eluting at closed arrow; 5- KtrA_ΔC_+KtrB with ATP eluting at open arrow; 6- Pure KtrB. Inset in **b)** 1- Pure KtrB_R417K_; 2- Pure KtrB_R417K_; 3- KtrA+KtrB_R417K_ with ADP eluting at closed arrow; 4- KtrA+KtrB_R417K_ with ADP eluting at open arrow; 5- KtrA+KtrB_R417K_ with ATP eluting at closed arrow; 6- KtrA+KtrB_R417K_ with ATP eluting at open arrow. Lower horizontal arrow indicates KtrA_ΔC_-tandem or full-length KtrA; horizontal arrows with M or D indicate KtrB monomer or dimer, respectively
(TIF)

S3 Fig Western blots of fractions collected from float-up experiments. Westerns were probed with anti-KtrB or anti-KtrA antibody. Fractions 1 to 5 correspond to 40 μl fractions collected from low to high sucrose concentration; liposomes float to the low density fractions. KtrB float-up distribution for **a)** KtrAB-ATP and -ADP reconstituted liposomes; or b) KtrA_ΔC_B-ATP and -ADP reconstituted liposomes. The experiments show that reconstituted KtrB is present in fractions 1 and 2 demonstrating that the membrane protein is associated with liposomes. KtrA float-up distribution after external addition of KtrA-ATP or -ADP to preformed **c)** empty liposomes or **d)** KtrB-reconstituted liposomes. Importantly, just like in the flux assay, KtrA was added in excess relative to KtrB. In the liposomes without KtrB (empty liposomes), the KtrA protein is not associated with liposomes and is detected in the high density fractions. When KtrA was added to KtrB liposomes, the protein distribution is changed such that KtrA is detected in all fractions, some is associated with the liposomes, and some is present as free protein in the fractions with a high concentration of sucrose. L indicates sample loaded in float-up, + indicates sample before reconstitution. Horizontal arrows with M or D indicate KtrB monomer or dimer, respectively.
(TIF)

S4 Fig Contents of asymmetric unit. Contents of asymmetric unit in KtrA_ΔC_B crystals are shown, with two KtrB dimers associated with one KtrA_ΔC_ ring on opposite faces. Subunits are shown in different colors. Spheres indicate K^+^.
(TIF)

S5 Fig Collection of KtrA RCK rings. RCK rings (without C-terminal domain) used as molecular search models against KtrAB-ADP data from: **a)** full-length KtrA-ATP (model 1—PDB code 4J90); **b)** full-length KtrA-ADP (model 2—PDB code 4J91); **c)** KtrA_ΔC_ (model 3—PDB code 2HMS); **d)** KtrA_ΔC_ (model 4—PDB code 2HMW); **e)** KtrA_ΔC_ (model 5—PDB code 2HMU); f) KtrA_ΔC_ from KtrA_ΔC_B structure (model 6 and 7—PDB code 5BUT). F71-Cα atoms at each of the subunits are shown as black spheres. Distances between F71 in pairs of opposite subunits are indicated for each ring in Angstroms.
(TIF)

S6 Fig Averaged map of KtrAB-ADP. Averaged map calculated with KtrAB-ADP 8 Å diffraction data and using starting phases calculated from the model 7 molecular replacement solution (S3 Table). Superposed on the map is the KtrB model from KtrA_ΔC_B and the full-length KtrA-ADP structure previously determined. Two KtrB dimers are indicated with density covering one of the dimers. Density covering the N-terminal domain and C-terminal domain (not included in the initial phasing) of two KtrA subunits is also indicated.
(TIF)

S7 Fig Comparison of RCK rings in KtrA_ΔC_B and KtrA-ADP. **a)** Face view of superposed KtrA-ADP (black Cα trace) and KtrA_ΔC_ (cyan). F71-Cα atoms are shown as cyan and black spheres. Superposition (through one of the N-terminal domains) of KtrA_ΔC_ ring dimer (cyan) with dimer from **b)** KtrA-ADP (black) or **c)** KtrA-ATP (red) from KtrAB-ATP structure. C-terminal domains are not shown. Angle values referring to the structural changes between RCK ring dimers, and quoted in the main text, were measured between axes of the last α-helix in the N-terminal domain, as indicated by cyan, black, and red lines.
(TIF)

S8 Fig Movement of M1D2 helix. Averaged density (mesh) of M1D2 helix in two different KtrB subunits. Together with Fig 2D and 2E, these two panels show the averaged density for all M1D2 helices of the 4 KtrB subunits in the asymmetric unit of KtrA_ΔC_B. KtrAB-ATP M1D2 helices are shown in red (on the left) and KtrA_ΔC_B-ADP M1D2 helices are in cyan (on the right). Arrow indicates shift applied to cytosolic end of helix to bring it into density.
(TIF)

S9 Fig KtrA_ΔC_B Difference map. Fo-Fc difference map calculated with structure factors from KtrA_ΔC_B dataset and structure factors and phases from molecular replacement solution after rigid body refinement. Red mesh indicates negative density at 3 sigma contour level; green mesh indicates positive density at 3 sigma contour level. Initial model is shown superposed with density. The largest red mesh peaks correspond to the cytosolic end of M1D2 helix and part of the M1D3 helix indicating the model needs adjustments in these regions.
(TIF)

S10 Fig Control experiments for cysteine residues introduced in D1-D2 domain. **a)** Q115C, **b)** V126C, and **d)** V130C. Size exclusion chromatography profiles (left) and ^86^Rb^+^ uptake (center) values at 0 and 60 min of KtrAB complexes formed by KtrA_C0_ and KtrB mutants with ATP and ADP. For V126C, the time course of modification by DTNB is also shown (right); for the other mutants, the equivalent data is presented in the main text. Closed arrowhead indicates elution volume of KtrAB complex; open arrowhead indicates elution volume of individual components. All mutants form a complex with KtrA_C0_ in the presence of ATP or ADP, and retain the functional stimulation by ATP relative to ADP. SDS-PAGE of size-exclusion fractions are shown as insets. Inset in **a)** 1- KtrA+KtrB_Q115C_ with ADP eluting at closed arrow; 2- KtrA+KtrB_Q115C_ with ADP eluting at open arrow; 3- KtrA+KtrB_Q115C_ with ATP eluting at closed arrow; 4- KtrA+KtrB_Q115C_ with ATP eluting at open arrow; Inset in **b)** 1- KtrA+KtrB_V126C_ with ADP eluting at closed arrow; 2- KtrA+KtrB_V126C_ with ADP eluting at open arrow; 3- sample buffer; 4- KtrA+KtrB_V126C_ with ATP eluting at closed arrow; 5- KtrA+KtrB_V126C_ with ATP eluting at open arrow. Inset in **c)** 1- pure KtrBV130C; 2- KtrA+KtrB_V130C_ with ADP eluting at closed arrow; 3- KtrA+KtrB_V130C_ with ADP eluting at open arrow; 4- KtrA+KtrB_V130C_ with ATP eluting at closed arrow; 5- KtrA+KtrB_V130C_ with ATP eluting at open arrow. Lower horizontal arrow indicates KtrA; horizontal arrow with M indicates KtrB monomer. Numerical values are included in S1 Data.
(TIF)

S11 Fig Control experiments for cytosolic pore cysteines. **a)** N119C, **b)** P121C, **d)** F443C and **e)** T444C. Size exclusion chromatography profiles (left) and ^86^Rb^+^ uptake (right) values at 0 and 60 min of KtrAB complexes formed by KtrA_C0_ and KtrB mutants with ATP and ADP. Closed arrowhead indicates elution volume of KtrAB complex; open arrowhead indicates elution volume of individual components. All mutants form a complex with KtrA_C0_ in the presence of ATP or ADP and retain the functional stimulation by ATP relative to ADP, although reduced in F443C. SDS-PAGE of size-exclusion fractions are shown as insets. Inset in **a)** 1- KtrA+KtrB_N119C_ with ADP eluting at closed arrow; 2- KtrA+KtrB_N119C_ with ADP eluting at open arrow; 3- KtrA+KtrB_N119C_ with ATP eluting at closed arrow; 4- KtrA+KtrB_N119C_ with ATP eluting at open arrow; Inset in **b)** 1- pure KtrB_P121C_. 2- KtrA+KtrB_P121C_ with ADP eluting at closed arrow; 3- KtrA+KtrB_P121C_ with ADP eluting at open arrow; 4- KtrA+KtrB_P121C_ with ATP eluting at closed arrow; 5- KtrA+KtrB_P121C_ with ATP eluting at open arrow. Inset in **c)** 1- pure KtrB_F443C_. 2- KtrA+KtrB_F443C_ with ADP eluting at closed arrow; 3- KtrA+KtrB_F443C_ with ADP eluting at open arrow; 4- KtrA+KtrB_F443C_ with ATP eluting at closed arrow; 5- KtrA+KtrB_F443C_ with ATP eluting at open arrow. Inset in **d)** 1- pure KtrA; 2- pure KtrA; 3- pure KtrB_T444C_; 4- KtrA+KtrB_T444C_ with ADP eluting at closed arrow; 5- KtrA+KtrB_T444C_ with ATP eluting at closed arrow. Lower horizontal arrow indicates KtrA; horizontal arrow with M indicates KtrB monomer. Numerical values are included in S1 Data.
(TIF)

S12 Fig SDS-PAGE of size-exclusion fractions of KtrB_Δloop_, KtrB_G306S_, and KtrB_S309D_. **a)** KtrB_Δloop_. Lanes 1 to 3 correspond to 1 ml fractions from chromatography of KtrA+KtrB_Δloop_ with ADP (Fig 5A) collected around open arrow peak. Lanes 4 to 8 correspond to 1 ml fractions from chromatography of KtrA+KtrB_Δloop_ with ATP (Fig 5A), starting at closed arrow and covering the open arrow peak. **b)** KtrB_G306S_. 1- pure KtrA; 2- KtrB_G306S_ before size-exclusion; 3- KtrA+ KtrB_G306S_ with ADP at open arrow; 4- KtrA+ KtrB_G306S_ with ATP at open arrow. **c)** KtrB_S309D_. 1- pure KtrA; 2- KtrB_S309D_ before size-exclusion; 3- KtrA+ KtrB_S309D_ with ADP at open arrow; 4- KtrA+ KtrB_S309D_ with ATP at open arrow. Lower horizontal arrow indicates KtrA; horizontal arrow with M indicates KtrB monomer.
(TIF)

S13 Fig Western blots of fractions collected from float-up experiments. Westerns were probed with anti-KtrB antibody. Fractions 1 to 5 correspond to 40 μl fractions collected from low to high sucrose concentration with liposomes floating to the low density fractions. KtrB float-up distribution for **a)** wild type KtrB and KtrB_Δloop_ reconstituted liposomes; **b)** wild type KtrB and KtrB_R417K_ reconstituted liposomes or **c)** KtrAB_R417K_-ATP and -ADP reconstituted liposomes. The membrane protein is detected in the low sucrose concentration fractions, showing that it is associated with the liposomes. Horizontal arrows with M or D indicate KtrB monomer or dimer, respectively.
(TIF)

S1 Table Averaged absolute ^86^Rb^+^ uptake levels (counts per minute) of functional assays depicted in Fig 1. Tables show the averaged raw data (before valinomycin normalization) collected for the KtrA_∆C_B and KtrAB functional assays plotted in Fig 1A and 1B, respectively. The increased background values seen in the KtrA_∆C_B functional assay are explained by the much lower valinomycin values for the Control time course in KtrA_∆C_B (~26,000 cpm) relative to the same values in KtrAB (~54,000 cpm). The valinomcyin values are obtained at the end of the time course and are used for normalization of the Rb^+^ uptake, as a consequence normalized values for the KtrA_∆C_B control are larger than for the KtrAB control. It is important to realize that in all biochemical and functional assays, we use an excess of RCK ring to favor the formation of the KtrAB complex, which has 1 dimer and 1 ring and minimize the formation of the complex with 1 RCK ring and 2 dimers of KtrB (see S4 Fig). In these circumstances, control liposomes were formed in the presence of KtrA_ΔC_ and contain a large amount of free ring; in contrast, in liposomes reconstituted with KtrA_ΔC_B, a large fraction of the ring is involved in the formation of the complex. We do not know why KtrA_ΔC_ appears to increase leakiness while full-length KtrA does not. The normalized data plotted in Fig 1 are slightly different from normalized values calculated with data shown in S1 Table. For Fig 1, we first normalized each individual time course using the corresponding valinomycin value and then calculated the average for each timepoint. In S1 Table, the values for a particular time point or valinomycin addition are the average of different sample preparations, reconstitutions, and time courses prior to normalization.
(DOCX)

S2 Table Diffraction data and refinement statistics. Rmsd: root-mean-square deviation; values in parenthesis correspond to highest resolution bin.
(DOCX)

S3 Table Log of likelihood values for molecular replacement analysis. *- Incorrect packing between KtrB and the octameric ring. Molecular replacement functions for the wild-type KtrAB-ADP 8 Å diffraction dataset were calculated with PHASER and search models composed by KtrB homodimers from either the KtrAB-ATP or KtrA_ΔC_B-ADP structures and KtrA octameric rings adopting different conformations (after removing their C-terminal domains). The procedure involved sequential searches with the membrane protein and the gating ring; for many of these pairs, we were able to obtain molecular replacement solutions that display the expected packing between the membrane protein dimer and the gating ring. The values for the LLG function are a measure of how well the structural model agrees with the data. The pair formed by the KtrB homodimer and KtrA_ΔC_ ring both from KtrA_ΔC_B-ADP has the highest LLG value (LLG = 515) and therefore appears to fit the data better than the other models. To evaluate the sensitivity of LLG parameter to small improvements in the KtrB model (in particular, the capacity of LLG to distinguish the goodness of fit between search models 6 and 7), we performed a series of tests. We first distorted the KtrB search model from KtrA_ΔC_B-ADP with a 10° tilt (see below explanation for this tilt) of the cytosolic halves of the M1D1 (residues 15 to 29) or M1D3 (residues 227–241) helices. This conformational change has not been observed in any of the existing structures, and so with this tilting the new KtrB search models are distorted (worsened) relative to KtrA_ΔC_B-ADP and KtrAB-ATP. Molecular replacement searches were performed with PHASER using the distorted KtrB dimers together with KtrA_ΔC_ against the KtrAB-ADP 8 Å data. If the LLG parameter calculated in the search is sensitive to a distortion affecting 15 residues, then its values should be lower than 515, the value found for the final refined model of KtrA_ΔC_B-ADP; LLG for the model distorted at M1D1 was 506, and at M1D3 it was 503. For both cases, the packing of the different components was correct. This demonstrates that LLG is sensitive even to relatively small distortions of the search model. It also shows that even small changes in LLG (in these cases changes of less than 10%) indicate a model that is a worse or better fit to the data. We then altered KtrB of KtrAB-ATP. We changed the cytosolic half (residues128 to 140) of the M1D2 helix in so that it coincides with the helices of the KtrB search model in KtrA_ΔC_B-ADP. We called this new model KtrB_**improved**_. We performed the inverse operation on the KtrB search model of KtrA_ΔC_B-ADP so that it resembles KtrAB-ATP and called it KtrB_**worse**_. These changes corresponded to a 10–11° tilt of the cytosolic ends of the M1D2 helices around a pivot point, residue 140. Once again, if LLG is sensitive to these changes, then molecular replacement searches using KtrB_**improved**_ (together with KtrA_ΔC_) against the KtrAB-ADP 8 Å data should show an increase in LLG relative to the unmodified model, while KtrB_**worse**_ should result in a decrease in LLG relative to the unmodified model. KtrB_**improved**_ LLG went up from 473 to 496 while for KtrB_**worse**_ LLG went down from 515 to 496. Note also that the LLG value for KtrB_**improved**_ is still lower than 515, the value obtained with the components of KtrA_ΔC_B; this shows that besides the difference in the M1D2 helices, there are many other small adjustments that occurred during refinement of KtrA_ΔC_B-ADP. These small adjustments improved the model and made it even more like KtrAB-ADP so that the LLG value is the highest. Overall, these experiments demonstrate how sensitive LLG in Phaser is to the goodness of fit of a search model to a crystal structure. Moreover, they demonstrate that the difference between 473 and 515 shown in the two bottom searches listed on S3 Table is significant, supporting our conclusion that the conformation of both the membrane protein and the RCK ring in full-length KtrAB-ADP is similar to the conformations observed in our KtrA_ΔC_B-ADP structure.
(DOCX)

## Abbreviations

ADP: adenosine diphosphate
cpm.: counts per minute
DEN: Deformable Elastic Network
DTNB: 5,5’-dithio-bis(2-nitrobenzoic acid)
KtrA_ΔC_: KtrA protein with C-terminal domain truncated
LLG: log-of-likelihood
M1D2: M1 transmembrane helix in repeat D2
RCK: regulate conductance of K^+^
SEM: standard error of the mean
TNB: thio-nitrobenzoate
