# Supplementary figures and images for: Dissecting the Molecular Mechanism of Nucleotide-Dependent Activation of the KtrAB K+ Transporter
Source: PLoS Biol. 2016 Jan 15;14(1):e1002356. doi: 10.1371/journal.pbio.1002356 (PMC4714889; doi:10.1371/journal.pbio.1002356)

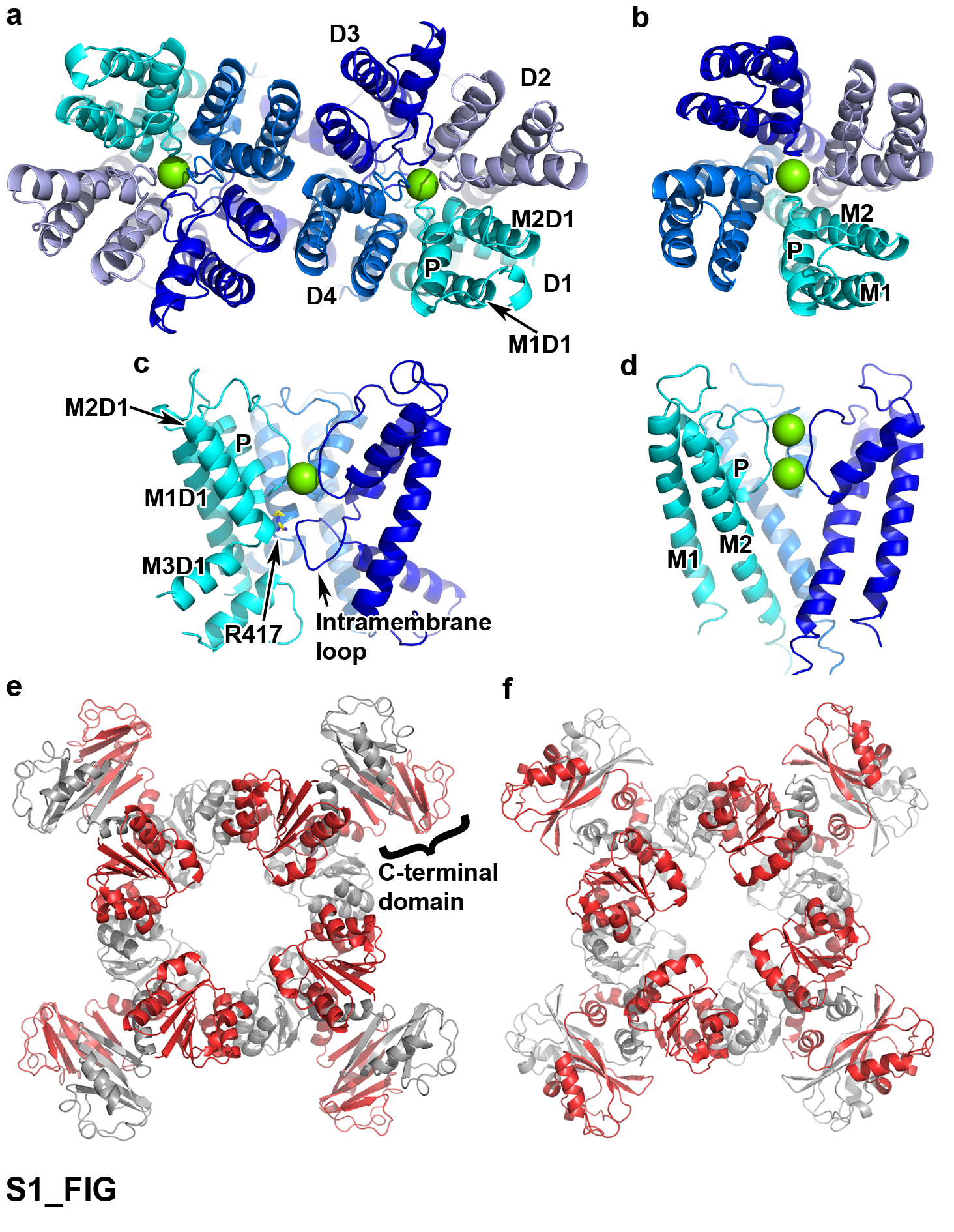

Supplement: S1 Fig — Extracellular views of a) KtrB homodimer and b) KcsA K+ channel. Side views of c) a single KtrB subunit without the 2nd repeat and d) KcsA pore without a channel subunit. Each repeat and subunit is colored a different shade of blue. KtrB repeats are labeled. Transmembrane and pore helices of one repeat or subunit are labeled. The intramembrane loop and conserved arginine are indicated. K+ are shown as green spheres bound in the selectivity filter. Structure of RCK octameric ring in e) KtrA (with a C-terminal domain indicated) and in f) MthK K+ channel. Alternate subunits are shown in red and gray. (TIF) [file pbio.1002356.s002.tif]

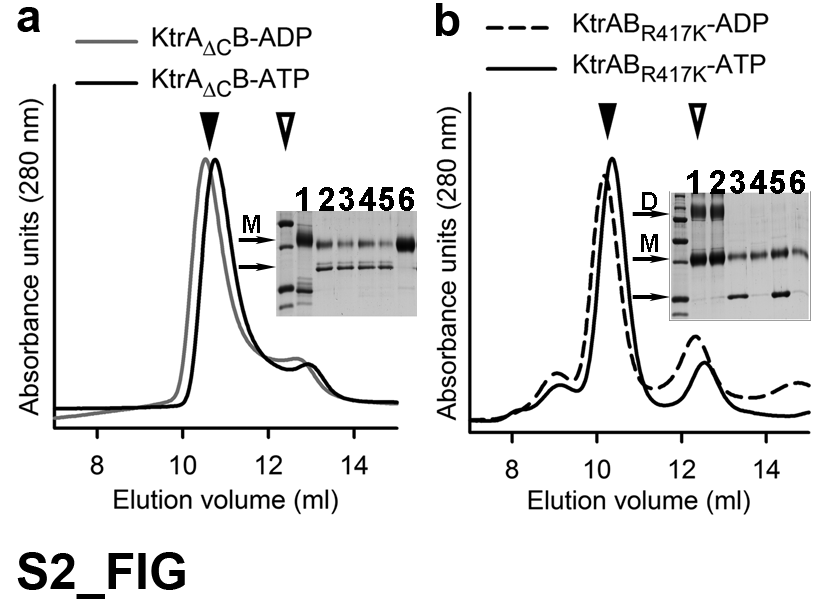

Supplement: S2 Fig — Size-exclusion profiles of a) KtrB assembled with KtrAΔC in the presence of ATP and ADP and b) KtrBR417K mutant assembled with KtrA in the presence of ATP and ADP. Closed inverted arrowhead indicates elution volume of KtrAB complex; open arrowhead indicates elution volume of individual components. SDS-PAGE of size-exclusion fractions are shown as insets. Inset in a) 1- KtrB preparation before size exclusion; 2- KtrAΔC+KtrB with ADP eluting at closed arrow; 3- KtrAΔC+KtrB with ADP eluting at open arrow; 4- KtrAΔC+KtrB with ATP eluting at closed arrow; 5- KtrAΔC+KtrB with ATP eluting at open arrow; 6- Pure KtrB. Inset in b) 1- Pure KtrBR417K; 2- Pure KtrBR417K; 3- KtrA+KtrBR417K with ADP eluting at closed arrow; 4- KtrA+KtrBR417K with ADP eluting at open arrow; 5- KtrA+KtrBR417K with ATP eluting at closed arrow; 6- KtrA+KtrBR417K with ATP eluting at open arrow. Lower horizontal arrow indicates KtrAΔC-tandem or full-length KtrA; horizontal arrows with M or D indicate KtrB monomer or dimer, respectively (TIF) [file pbio.1002356.s003.tif]

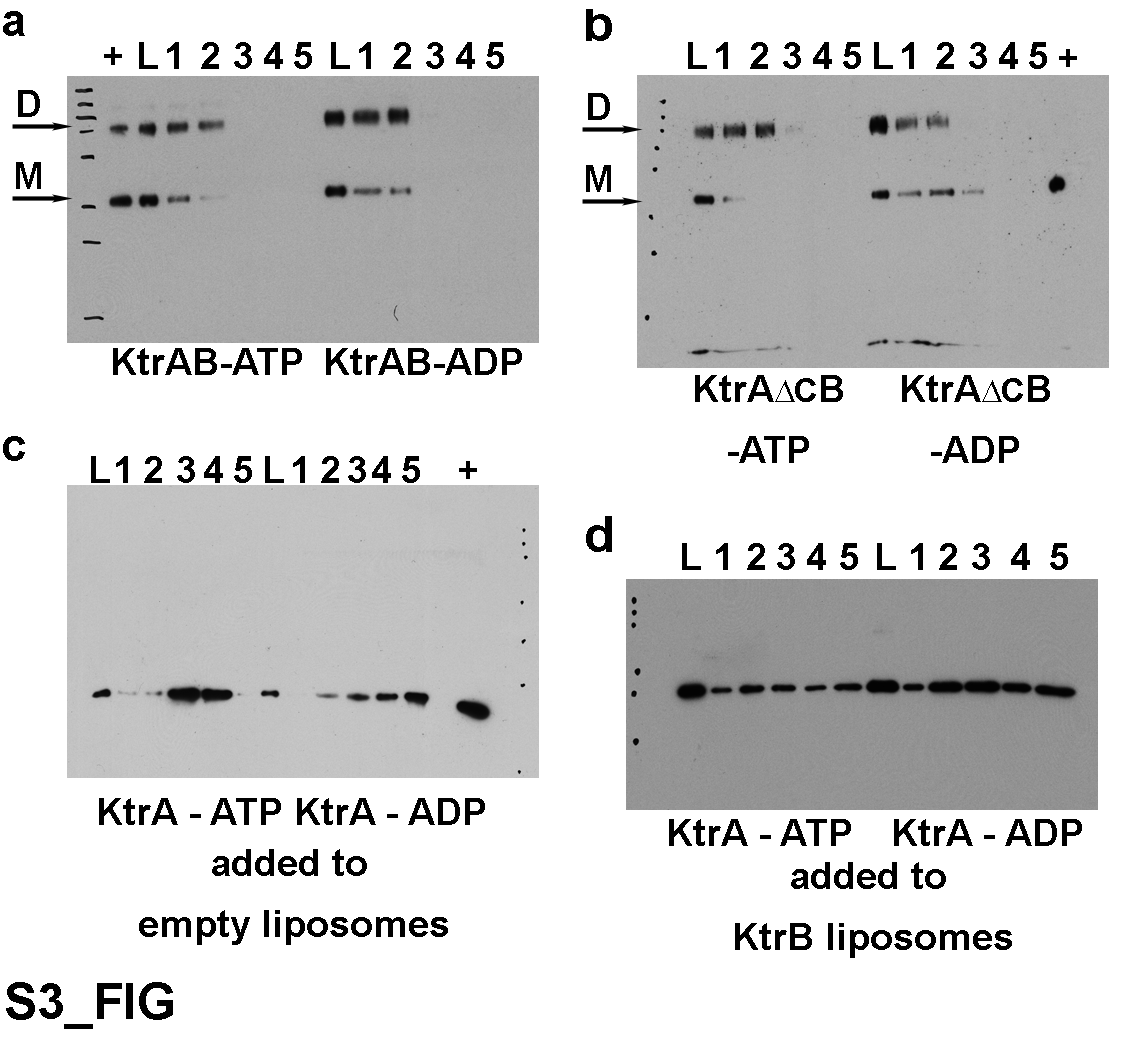

Supplement: S3 Fig — Westerns were probed with anti-KtrB or anti-KtrA antibody. Fractions 1 to 5 correspond to 40 μl fractions collected from low to high sucrose concentration; liposomes float to the low density fractions. KtrB float-up distribution for a) KtrAB-ATP and -ADP reconstituted liposomes; or b) KtrAΔCB-ATP and -ADP reconstituted liposomes. The experiments show that reconstituted KtrB is present in fractions 1 and 2 demonstrating that the membrane protein is associated with liposomes. KtrA float-up distribution after external addition of KtrA-ATP or -ADP to preformed c) empty liposomes or d) KtrB-reconstituted liposomes. Importantly, just like in the flux assay, KtrA was added in excess relative to KtrB. In the liposomes without KtrB (empty liposomes), the KtrA protein is not associated with liposomes and is detected in the high density fractions. When KtrA was added to KtrB liposomes, the protein distribution is changed such that KtrA is detected in all fractions, some is associated with the liposomes, and some is present as free protein in the fractions with a high concentration of sucrose. L indicates sample loaded in float-up, + indicates sample before reconstitution. Horizontal arrows with M or D indicate KtrB monomer or dimer, respectively. (TIF) [file pbio.1002356.s004.tif]

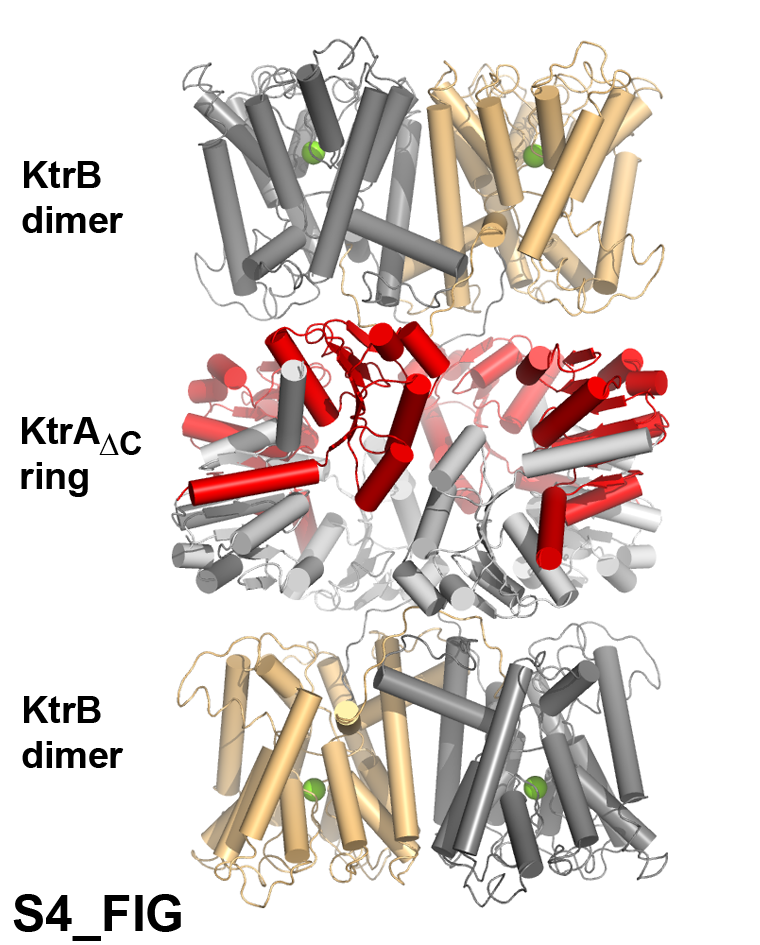

Supplement: S4 Fig — Contents of asymmetric unit in KtrAΔCB crystals are shown, with two KtrB dimers associated with one KtrAΔC ring on opposite faces. Subunits are shown in different colors. Spheres indicate K+. (TIF) [file pbio.1002356.s005.tif]

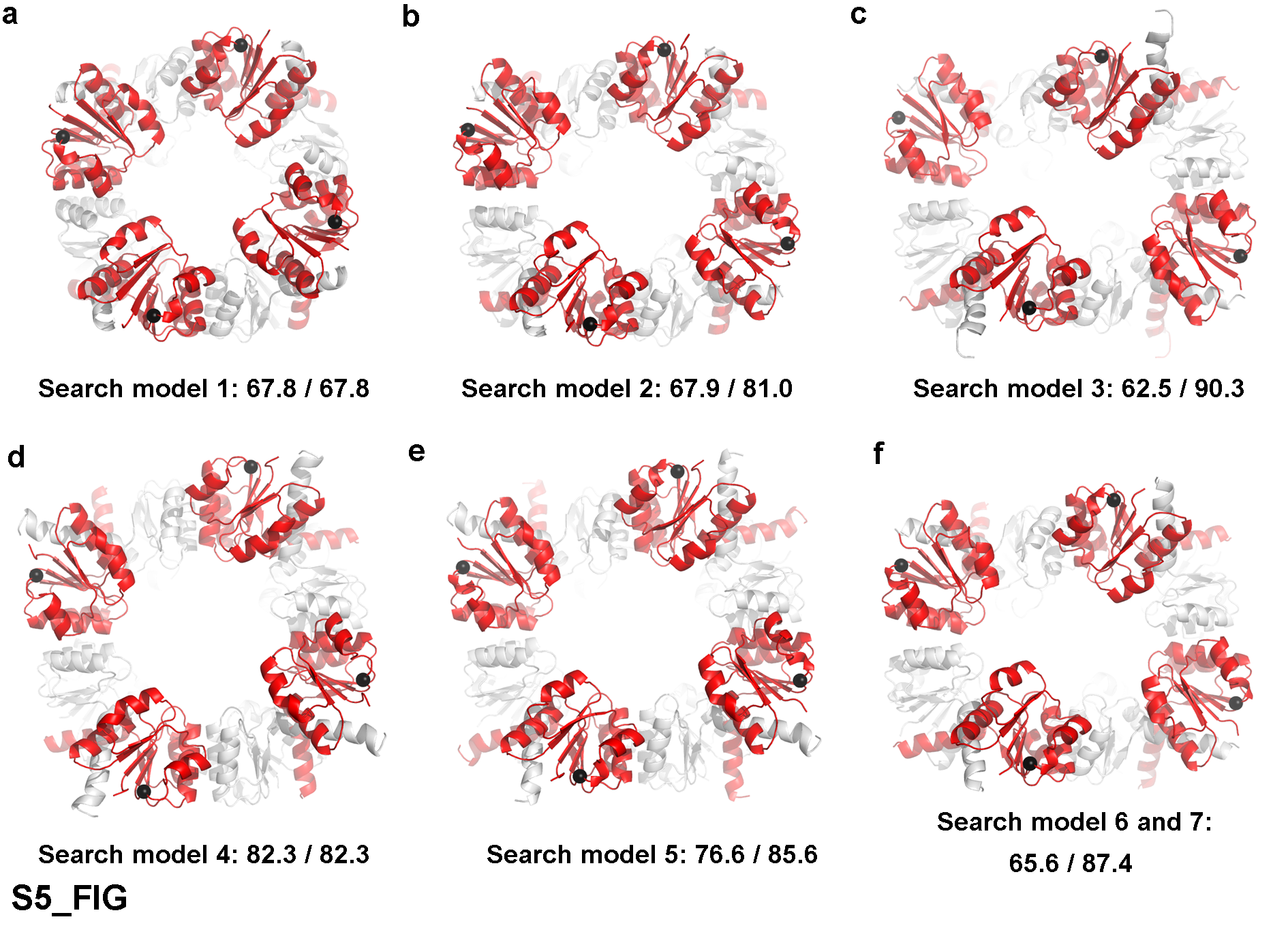

Supplement: S5 Fig — RCK rings (without C-terminal domain) used as molecular search models against KtrAB-ADP data from: a) full-length KtrA-ATP (model 1—PDB code 4J90); b) full-length KtrA-ADP (model 2—PDB code 4J91); c) KtrAΔC (model 3—PDB code 2HMS); d) KtrAΔC (model 4—PDB code 2HMW); e) KtrAΔC (model 5—PDB code 2HMU); f) KtrAΔC from KtrAΔCB structure (model 6 and 7—PDB code 5BUT). F71-Cα atoms at each of the subunits are shown as black spheres. Distances between F71 in pairs of opposite subunits are indicated for each ring in Angstroms. (TIF) [file pbio.1002356.s006.tif]

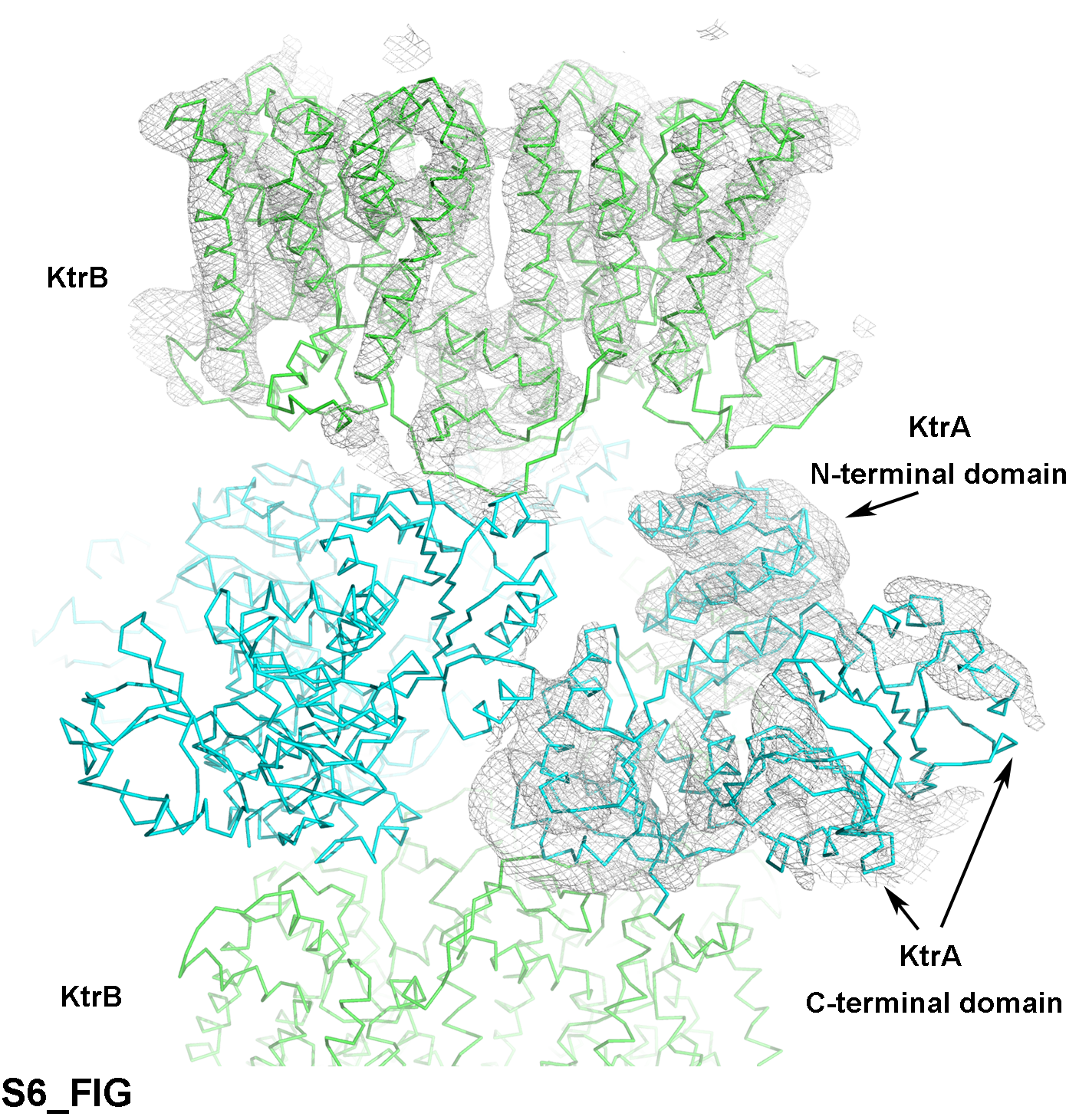

Supplement: S6 Fig — Averaged map calculated with KtrAB-ADP 8 Å diffraction data and using starting phases calculated from the model 7 molecular replacement solution (S3 Table). Superposed on the map is the KtrB model from KtrAΔCB and the full-length KtrA-ADP structure previously determined. Two KtrB dimers are indicated with density covering one of the dimers. Density covering the N-terminal domain and C-terminal domain (not included in the initial phasing) of two KtrA subunits is also indicated. (TIF) [file pbio.1002356.s007.tif]

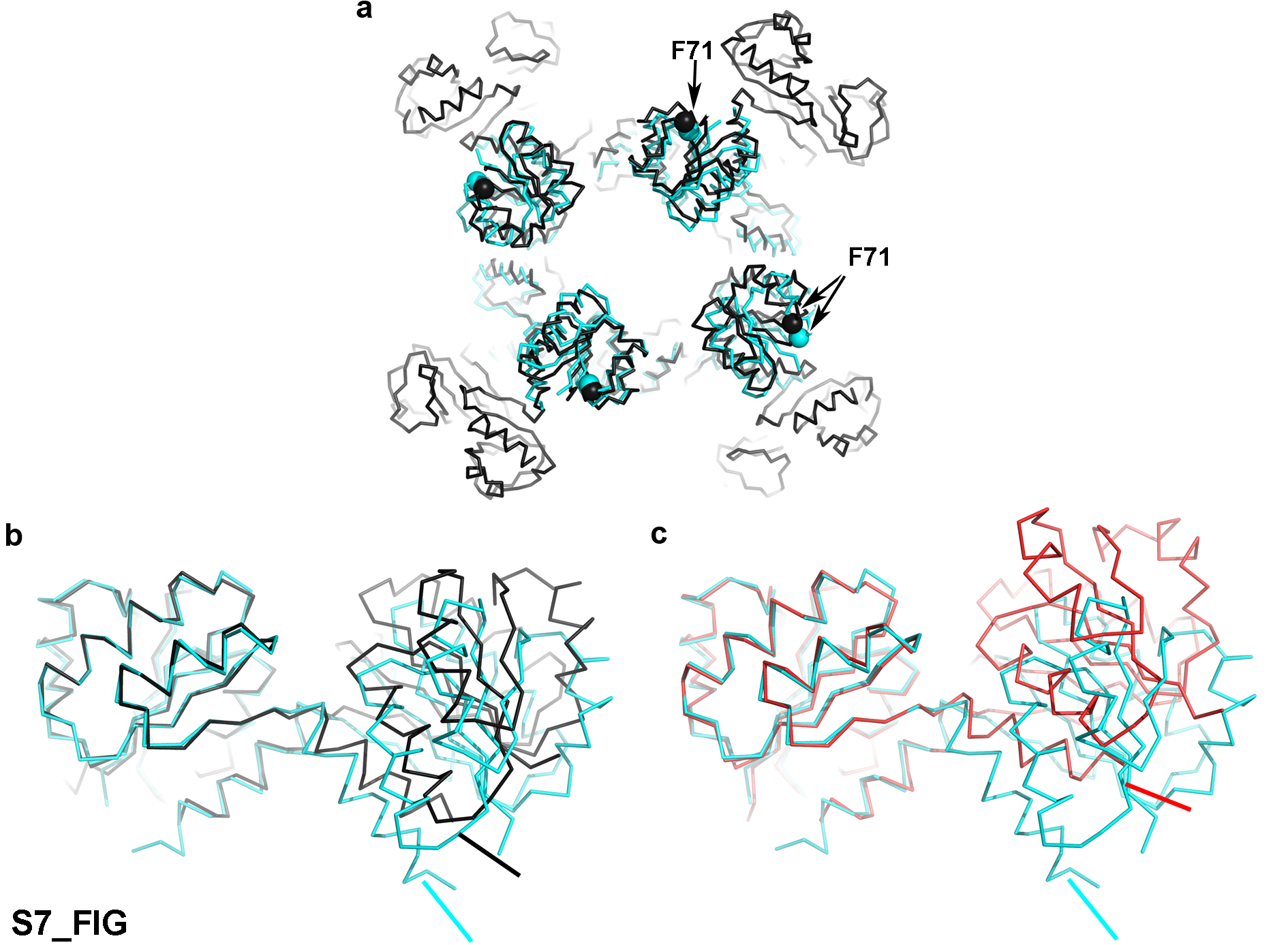

Supplement: S7 Fig — a) Face view of superposed KtrA-ADP (black Cα trace) and KtrAΔC (cyan). F71-Cα atoms are shown as cyan and black spheres. Superposition (through one of the N-terminal domains) of KtrAΔC ring dimer (cyan) with dimer from b) KtrA-ADP (black) or c) KtrA-ATP (red) from KtrAB-ATP structure. C-terminal domains are not shown. Angle values referring to the structural changes between RCK ring dimers, and quoted in the main text, were measured between axes of the last α-helix in the N-terminal domain, as indicated by cyan, black, and red lines. (TIF) [file pbio.1002356.s008.tif]

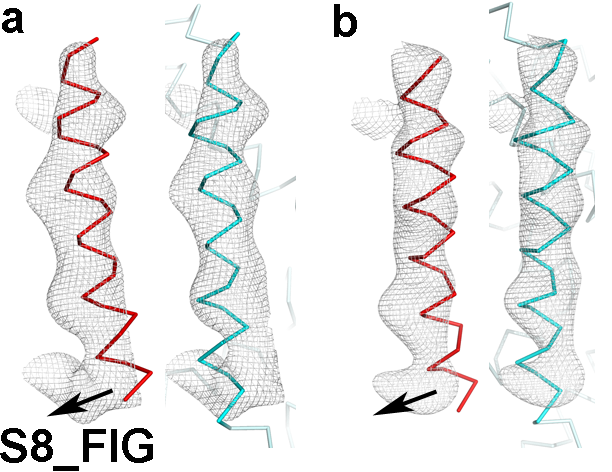

Supplement: S8 Fig — Averaged density (mesh) of M1D2 helix in two different KtrB subunits. Together with Fig 2D and 2E, these two panels show the averaged density for all M1D2 helices of the 4 KtrB subunits in the asymmetric unit of KtrAΔCB. KtrAB-ATP M1D2 helices are shown in red (on the left) and KtrAΔCB-ADP M1D2 helices are in cyan (on the right). Arrow indicates shift applied to cytosolic end of helix to bring it into density. (TIF) [file pbio.1002356.s009.tif]

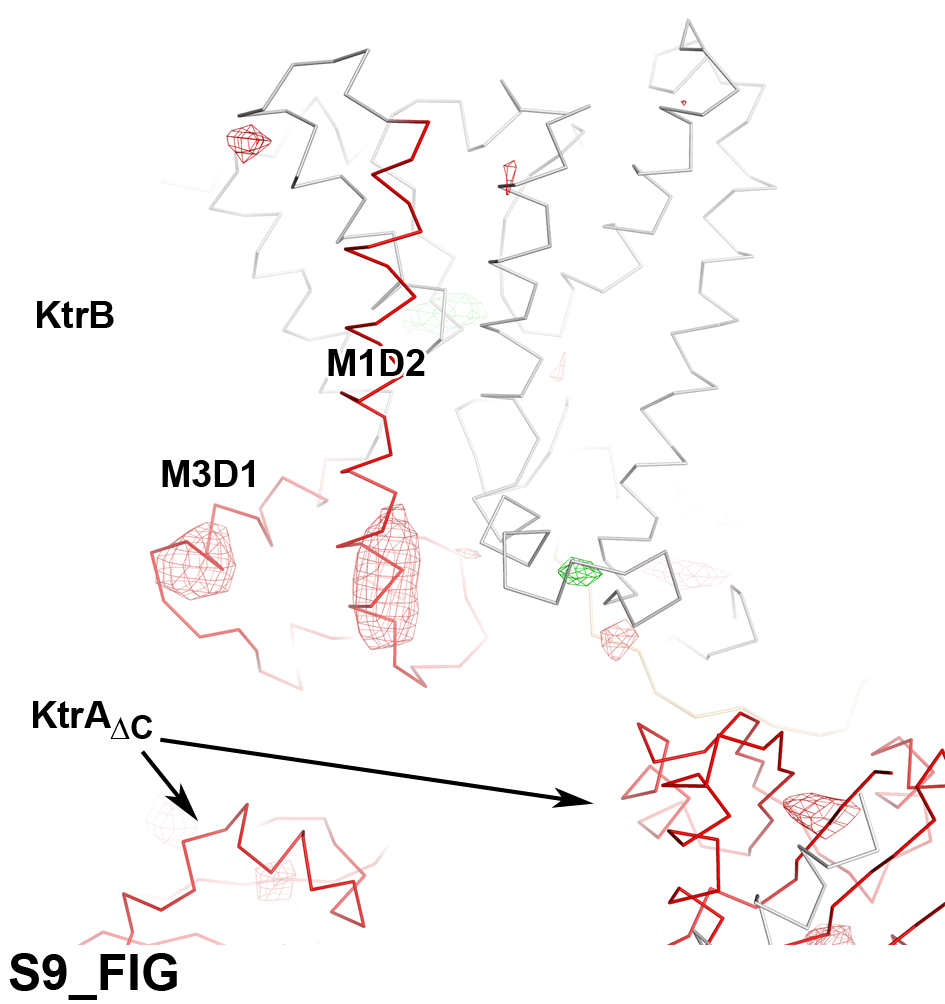

Supplement: S9 Fig — Fo-Fc difference map calculated with structure factors from KtrAΔCB dataset and structure factors and phases from molecular replacement solution after rigid body refinement. Red mesh indicates negative density at 3 sigma contour level; green mesh indicates positive density at 3 sigma contour level. Initial model is shown superposed with density. The largest red mesh peaks correspond to the cytosolic end of M1D2 helix and part of the M1D3 helix indicating the model needs adjustments in these regions. (TIF) [file pbio.1002356.s010.tif]

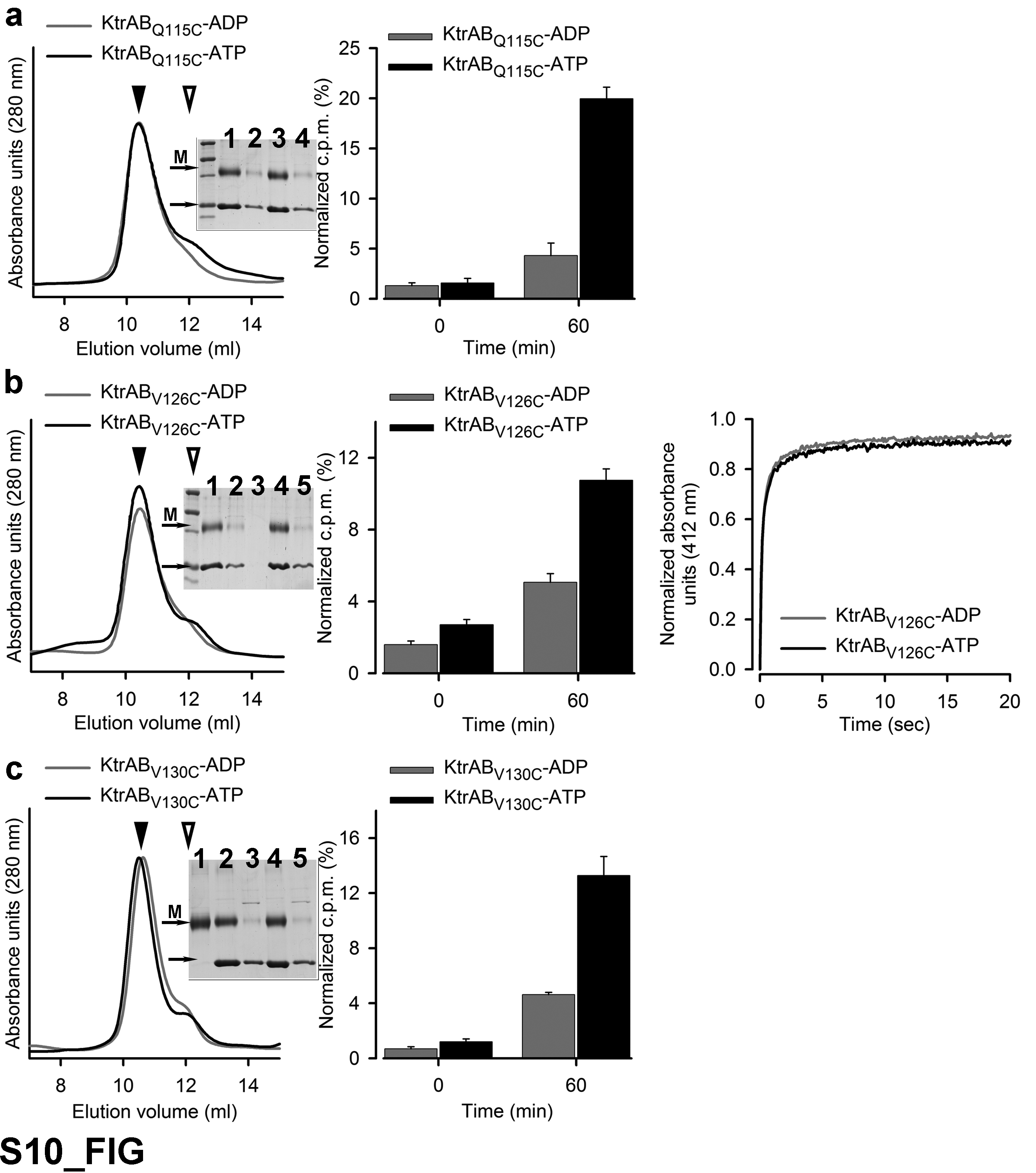

Supplement: S10 Fig — a) Q115C, b) V126C, and d) V130C. Size exclusion chromatography profiles (left) and 86Rb+ uptake (center) values at 0 and 60 min of KtrAB complexes formed by KtrAC0 and KtrB mutants with ATP and ADP. For V126C, the time course of modification by DTNB is also shown (right); for the other mutants, the equivalent data is presented in the main text. Closed arrowhead indicates elution volume of KtrAB complex; open arrowhead indicates elution volume of individual components. All mutants form a complex with KtrAC0 in the presence of ATP or ADP, and retain the functional stimulation by ATP relative to ADP. SDS-PAGE of size-exclusion fractions are shown as insets. Inset in a) 1- KtrA+KtrBQ115C with ADP eluting at closed arrow; 2- KtrA+KtrBQ115C with ADP eluting at open arrow; 3- KtrA+KtrBQ115C with ATP eluting at closed arrow; 4- KtrA+KtrBQ115C with ATP eluting at open arrow; Inset in b) 1- KtrA+KtrBV126C with ADP eluting at closed arrow; 2- KtrA+KtrBV126C with ADP eluting at open arrow; 3- sample buffer; 4- KtrA+KtrBV126C with ATP eluting at closed arrow; 5- KtrA+KtrBV126C with ATP eluting at open arrow. Inset in c) 1- pure KtrBV130C; 2- KtrA+KtrBV130C with ADP eluting at closed arrow; 3- KtrA+KtrBV130C with ADP eluting at open arrow; 4- KtrA+KtrBV130C with ATP eluting at closed arrow; 5- KtrA+KtrBV130C with ATP eluting at open arrow. Lower horizontal arrow indicates KtrA; horizontal arrow with M indicates KtrB monomer. Numerical values are included in S1 Data. (TIF) [file pbio.1002356.s011.tif]

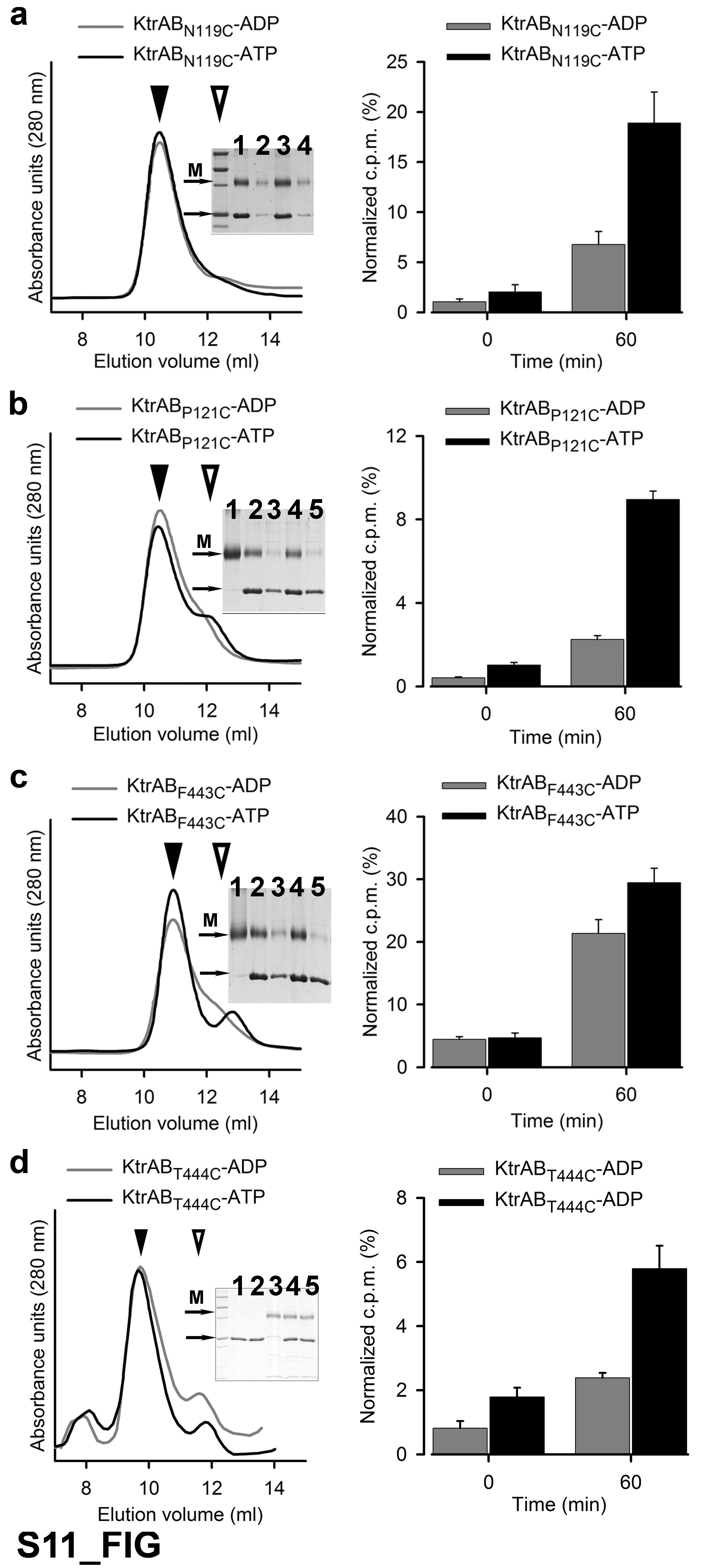

Supplement: S11 Fig — a) N119C, b) P121C, d) F443C and e) T444C. Size exclusion chromatography profiles (left) and 86Rb+ uptake (right) values at 0 and 60 min of KtrAB complexes formed by KtrAC0 and KtrB mutants with ATP and ADP. Closed arrowhead indicates elution volume of KtrAB complex; open arrowhead indicates elution volume of individual components. All mutants form a complex with KtrAC0 in the presence of ATP or ADP and retain the functional stimulation by ATP relative to ADP, although reduced in F443C. SDS-PAGE of size-exclusion fractions are shown as insets. Inset in a) 1- KtrA+KtrBN119C with ADP eluting at closed arrow; 2- KtrA+KtrBN119C with ADP eluting at open arrow; 3- KtrA+KtrBN119C with ATP eluting at closed arrow; 4- KtrA+KtrBN119C with ATP eluting at open arrow; Inset in b) 1- pure KtrBP121C. 2- KtrA+KtrBP121C with ADP eluting at closed arrow; 3- KtrA+KtrBP121C with ADP eluting at open arrow; 4- KtrA+KtrBP121C with ATP eluting at closed arrow; 5- KtrA+KtrBP121C with ATP eluting at open arrow. Inset in c) 1- pure KtrBF443C. 2- KtrA+KtrBF443C with ADP eluting at closed arrow; 3- KtrA+KtrBF443C with ADP eluting at open arrow; 4- KtrA+KtrBF443C with ATP eluting at closed arrow; 5- KtrA+KtrBF443C with ATP eluting at open arrow. Inset in d) 1- pure KtrA; 2- pure KtrA; 3- pure KtrBT444C; 4- KtrA+KtrBT444C with ADP eluting at closed arrow; 5- KtrA+KtrBT444C with ATP eluting at closed arrow. Lower horizontal arrow indicates KtrA; horizontal arrow with M indicates KtrB monomer. Numerical values are included in S1 Data. (TIF) [file pbio.1002356.s012.tif]

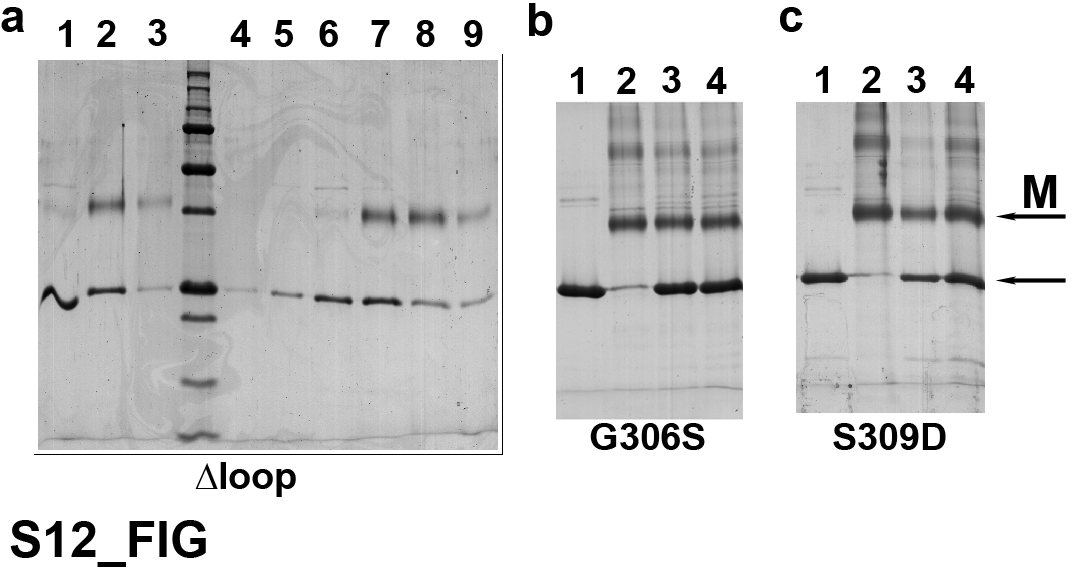

Supplement: S12 Fig — a) KtrBΔloop. Lanes 1 to 3 correspond to 1 ml fractions from chromatography of KtrA+KtrBΔloop with ADP (Fig 5A) collected around open arrow peak. Lanes 4 to 8 correspond to 1 ml fractions from chromatography of KtrA+KtrBΔloop with ATP (Fig 5A), starting at closed arrow and covering the open arrow peak. b) KtrBG306S. 1- pure KtrA; 2- KtrBG306S before size-exclusion; 3- KtrA+ KtrBG306S with ADP at open arrow; 4- KtrA+ KtrBG306S with ATP at open arrow. c) KtrBS309D. 1- pure KtrA; 2- KtrBS309D before size-exclusion; 3- KtrA+ KtrBS309D with ADP at open arrow; 4- KtrA+ KtrBS309D with ATP at open arrow. Lower horizontal arrow indicates KtrA; horizontal arrow with M indicates KtrB monomer. (TIF) [file pbio.1002356.s013.tif]

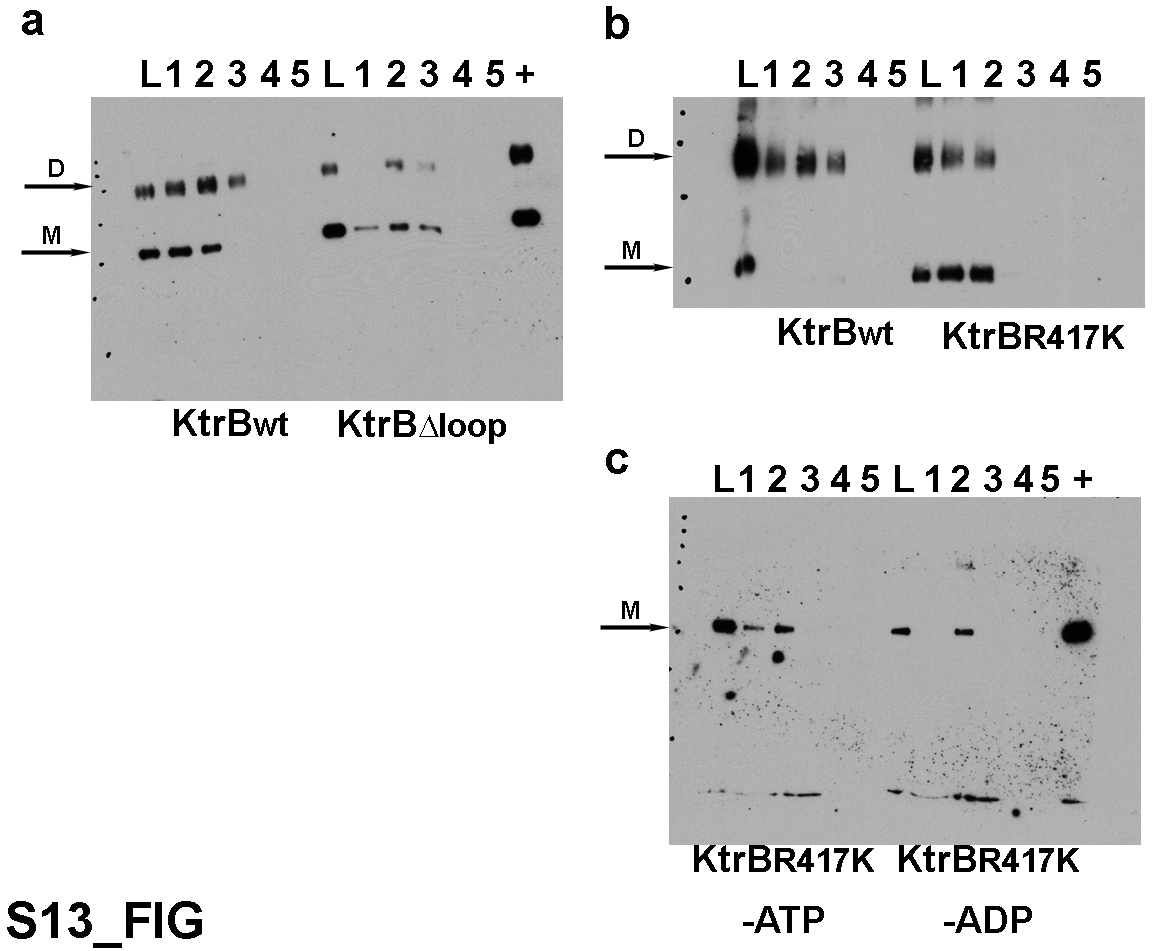

Supplement: S13 Fig — Westerns were probed with anti-KtrB antibody. Fractions 1 to 5 correspond to 40 μl fractions collected from low to high sucrose concentration with liposomes floating to the low density fractions. KtrB float-up distribution for a) wild type KtrB and KtrBΔloop reconstituted liposomes; b) wild type KtrB and KtrBR417K reconstituted liposomes or c) KtrABR417K-ATP and -ADP reconstituted liposomes. The membrane protein is detected in the low sucrose concentration fractions, showing that it is associated with the liposomes. Horizontal arrows with M or D indicate KtrB monomer or dimer, respectively. (TIF) [file pbio.1002356.s014.tif]
