# Supplementary material for: Dissecting the Molecular Mechanism of Nucleotide-Dependent Activation of the KtrAB K+ Transporter
Source: PLoS Biol. 2016 Jan 15;14(1):e1002356. doi: 10.1371/journal.pbio.1002356 (PMC4714889; doi:10.1371/journal.pbio.1002356)
Supplement: S1 Table — Tables show the averaged raw data (before valinomycin normalization) collected for the KtrA∆CB and KtrAB functional assays plotted in Fig 1A and 1B, respectively. The increased background values seen in the KtrA∆CB functional assay are explained by the much lower valinomycin values for the Control time course in KtrA∆CB (~26,000 cpm) relative to the same values in KtrAB (~54,000 cpm). The valinomcyin values are obtained at the end of the time course and are used for normalization of the Rb+ uptake, as a consequence normalized values for the KtrA∆CB control are larger than for the KtrAB control. It is important to realize that in all biochemical and functional assays, we use an excess of RCK ring to favor the formation of the KtrAB complex, which has 1 dimer and 1 ring and minimize the formation of the complex with 1 RCK ring and 2 dimers of KtrB (see S4 Fig). In these circumstances, control liposomes were formed in the presence of KtrAΔC and contain a large amount of free ring; in contrast, in liposomes reconstituted with KtrAΔCB, a large fraction of the ring is involved in the formation of the complex. We do not know why KtrAΔC appears to increase leakiness while full-length KtrA does not. The normalized data plotted in Fig 1 are slightly different from normalized values calculated with data shown in S1 Table. For Fig 1, we first normalized each individual time course using the corresponding valinomycin value and then calculated the average for each timepoint. In S1 Table, the values for a particular time point or valinomycin addition are the average of different sample preparations, reconstitutions, and time courses prior to normalization. (DOCX) [file pbio.1002356.s015.docx]

**S1_Table: Averaged absolute ^86^Rb^+^ uptake levels (c.p.m.) of functional assays depicted in Fig 1.**

**Averaged Raw Data for KtrA_∆C_B**

| **Time (min)** | **Control** | **KtrB** | **KtrA_∆C_B-ADP** | **KtrA_∆C_B-ATP** |
| --- | --- | --- | --- | --- |
| 0 | 483 ± 47 | 661 ± 50 | 826 ± 175 | 587 ± 47 |
| 5 | 772 ± 54 | 1329 ± 89 | 1248 ± 123 | 1230 ± 94 |
| 15 | 1179 ± 96 | 2092 ± 147 | 1890 ± 150 | 1829 ± 163 |
| 30 | 1596 ± 167 | 2558 ± 200 | 2374 ± 178 | 2571 ± 204 |
| 60 | 2389 ± 299 | 3311 ± 233 | 2852 ± 216 | 3042 ± 146 |
| Valinomycin | 26742 ± 3337 | 33881 ± 6690 | 23797 ± 4918 | 26497 ± 5574 |

**Averaged Raw Data for KtrAB**

| **Time (min)** | **Control** | **KtrB** | **KtrAB-ADP** | **KtrAB-ATP** |
| --- | --- | --- | --- | --- |
| 0 | 822 ± 167 | 1038 ± 132 | 926 ± 139 | 1076 ± 159 |
| 5 | 823 ± 214 | 1802 ± 167 | 1503 ± 170 | 1979 ± 304 |
| 15 | 924 ± 156 | 2468 ± 189 | 2036 ± 245 | 2769 ± 516 |
| 30 | 1031 ± 164 | 2905 ± 165 | 2470 ± 250 | 3339 ± 637 |
| 60 | 1027 ± 144 | 3150 ± 138 | 2869 ± 264 | 4158 ± 708 |
| Valinomycin | 54317 ± 2758 | 32499 ± 2612 | 32876 ± 2494 | 21777 ± 1627 |
