# Supplementary material for: Dissecting the Molecular Mechanism of Nucleotide-Dependent Activation of the KtrAB K+ Transporter
Source: PLoS Biol. 2016 Jan 15;14(1):e1002356. doi: 10.1371/journal.pbio.1002356 (PMC4714889; doi:10.1371/journal.pbio.1002356)
Supplement: S2 Table — Rmsd: root-mean-square deviation; values in parenthesis correspond to highest resolution bin. (DOCX) [file pbio.1002356.s016.docx]

**S2_Table:** **Diffraction data and refinement statistics**

| **Crystal** | KtrA_ΔC_B-ADP | KtrAB-ADP |
| --- | --- | --- |
| Space group | C2 | P2_1_ |
| Unit cell parameters (Å)  (º) | 307.1, 79.4, 205.7  90, 98.1, 90 | 123.9, 157.9, 150.9  90, 110.4, 90 |
| **Data collection** | Soleil Proxima 2 | ESRF ID14-4 |
| Resolution range (Å) | 50 - 5.97 (6.3 - 5.97) | 49 - 7.8 (8.7 - 7.8) |
| Number of observations | 43649 | 23176 |
| Number of unique reflections | 12628 | 6278 |
| Completeness (%) | 98.2 (98.1) | 98.9 (98.1) |
| Mean I/σI | 10.2 (1.6) | 5.5 (1.1) |
| Rmerge on I (%) | 7.0 (67.1) | 12.5(118) |
| Rmeasure on I (%) | 8.3 (79.5) | 14.6 (138) |
| CC_1/2_ | 0.795 | 0.445 |
| **Model and refinement statistics for DEN refinement** | | |
| Resolution range (Å) | 58.4 – 5.98 (6.19- 5.98) | N/A |
| Ramachandran favored (%) | 80.4 |  |
| Ramachandran allowed (%) | 16.0 |  |
| Ramachandran outliers (%) | 3.6 |  |
| Bond angle rmsd (º) | 0.83 |  |
| Bond length rmsd (Å) | 0.0034 |  |
| B factor (Å^2^) | 343 |  |
| Number of residues | 2796 + 4 K^+^ |  |
| R_free_ (%) | 33.9 (50.3) |  |
| R_work_ (%) | 32.5 (45.4) |  |

Rmsd: root-mean-square deviation; values in parenthesis correspond to highest resolution bin.
