# Supplementary material for: Dissecting the Molecular Mechanism of Nucleotide-Dependent Activation of the KtrAB K+ Transporter
Source: PLoS Biol. 2016 Jan 15;14(1):e1002356. doi: 10.1371/journal.pbio.1002356 (PMC4714889; doi:10.1371/journal.pbio.1002356)
Supplement: S3 Table — *- Incorrect packing between KtrB and the octameric ring. Molecular replacement functions for the wild-type KtrAB-ADP 8 Å diffraction dataset were calculated with PHASER and search models composed by KtrB homodimers from either the KtrAB-ATP or KtrAΔCB-ADP structures and KtrA octameric rings adopting different conformations (after removing their C-terminal domains). The procedure involved sequential searches with the membrane protein and the gating ring; for many of these pairs, we were able to obtain molecular replacement solutions that display the expected packing between the membrane protein dimer and the gating ring. The values for the LLG function are a measure of how well the structural model agrees with the data. The pair formed by the KtrB homodimer and KtrAΔC ring both from KtrAΔCB-ADP has the highest LLG value (LLG = 515) and therefore appears to fit the data better than the other models. To evaluate the sensitivity of LLG parameter to small improvements in the KtrB model (in particular, the capacity of LLG to distinguish the goodness of fit between search models 6 and 7), we performed a series of tests. We first distorted the KtrB search model from KtrAΔCB-ADP with a 10° tilt (see below explanation for this tilt) of the cytosolic halves of the M1D1 (residues 15 to 29) or M1D3 (residues 227–241) helices. This conformational change has not been observed in any of the existing structures, and so with this tilting the new KtrB search models are distorted (worsened) relative to KtrAΔCB-ADP and KtrAB-ATP. Molecular replacement searches were performed with PHASER using the distorted KtrB dimers together with KtrAΔC against the KtrAB-ADP 8 Å data. If the LLG parameter calculated in the search is sensitive to a distortion affecting 15 residues, then its values should be lower than 515, the value found for the final refined model of KtrAΔCB-ADP; LLG for the model distorted at M1D1 was 506, and at M1D3 it was 503. For both cases, the packing of the different compone [file pbio.1002356.s017.docx]

**S3_Table: Log of likelihood values for molecular replacement analysis**

| **Search Models** | | **Log-likelihood** |
| --- | --- | --- |
| 1 | - KtrB dimer from KtrAB-ATP structure (PDB code: 4J7C)  - KtrA-ATP ring (PDB code: 4J90, without the C-terminal domain) | 223* |
| 2 | - KtrB dimer from KtrAB-ATP structure (PDB code: 4J7C)  - KtrA-ADP ring (PDB code: 4J91, without the C-terminal domain) | 288 |
| 3 | - KtrB dimer from KtrAB-ATP structure (PDB code: 4J7C)  - KtrA_ΔC_ (PDB code: 2HMS, rectangular shaped conformation) | 195* |
| 4 | - KtrB dimer from KtrAB-ATP structure (PDB code: 4J7C)  - KtrA_ΔC_ (PDB code: 2HMW, square shaped conformation) | 252 |
| 5 | - KtrB dimer from KtrAB-ATP structure (PDB code: 4J7C)  - KtrA_ΔC_ (PDB code: 2HMU, diamond shaped conformation) | 302 |
| 6 | - KtrB dimer from KtrAB-ATP structure (PDB code: 4J7C)  - KtrA_ΔC_ from KtrA_ΔC_B structure | 473 |
| 7 | - KtrB dimer from KtrA_ΔC_B structure  - KtrA_ΔC_ from KtrA_ΔC_B structure | 515 |

*- Incorrect packing between KtrB and the octameric ring
